# The effect of disease-modifying therapies on brain volume loss and disability accumulation in multiple sclerosis: a systematic review and network meta-analysis

**DOI:** 10.1016/j.lanepe.2025.101476

**Published:** 2025-09-27

**Authors:** Alessandro Cagol, Sabine Schaedelin, Roxanne Pretzsch, Ludwig Kappos, Maria Pia Sormani, Cristina Granziera

**Affiliations:** a Translational Imaging in Neurology (ThINk) Basel, Department of Biomedical Engineering, Faculty of Medicine, University Hospital Basel and University of Basel, Basel, Switzerland; b Multiple Sclerosis Centre, Departments of Neurology, Clinical Research and Biomedicine, University Hospital and University Basel, Switzerland; c Research Center for Clinical Neuroimmunology and Neuroscience Basel (RC2NB), University Hospital Basel and University of Basel, Basel, Switzerland; d Dipartimento di Scienze della Salute, Università degli Studi di Genova, Genova, Italy; e Department of Clinical Research, University Hospital Basel, University of Basel, Basel, Switzerland; f IRCCS Ospedale Policlinico San Martino, Genova, Italy

**Keywords:** Multiple sclerosis, Disease-modifying therapies, Brain volume loss, Randomized controlled trials, Network meta-analysis

## Abstract

**Background:**

Multiple treatments have demonstrated efficacy in preventing brain volume loss (BVL) in randomized controlled trials (RCTs) for multiple sclerosis (MS). However, assessing their relative effectiveness remains challenging due to limited head-to-head comparisons. Additionally, the relationship between treatment effects on BVL and disability accumulation is not established for newer therapies. This study aimed to compare the efficacy of approved disease-modifying therapies (DMTs) in reducing BVL in MS and to investigate the association between treatment effects on BVL and disability accumulation.

**Methods:**

In this systematic review and network meta-analysis, we included all RCTs enrolling adults with MS that evaluated FDA-approved DMTs and reported BVL outcomes over at least one year. We searched PubMed, Embase, and Cochrane from inception to September 2024. Following PRISMA guidelines, two reviewers independently extracted data on BVL, MRI lesion activity, and disability progression. We conducted a mixed-effects network meta-analysis with placebo as the reference group. Meta-regression analyses examined the association between treatment effects on BVL and disability progression, adjusting for MRI lesion activity.

The primary outcome was BVL. Secondary outcomes included MRI lesion accumulation and risk of confirmed disability progression. Effect sizes were reported as the ratio of means (ROM) and hazard ratios (HRs), with 95% confidence intervals (CIs). This study is registered with PROSPERO (CRD420251034936).

**Findings:**

We included 33 RCTs evaluating 16 DMTs and 26,247 patients. Eight DMTs significantly reduced BVL compared to placebo, including ponesimod (ROM = 0.52; 95%-CI: 0.35–0.77), ofatumumab (ROM = 0.58; 95%-CI: 0.40–0.83), alemtuzumab (ROM = 0.63; 95%-CI: 0.49–0.83), teriflunomide (ROM = 0.71; 95%-CI: 0.52–0.97), ozanimod (ROM = 0.74; 95%-CI: 0.56–0.98), natalizumab (ROM = 0.77; 95%-CI: 0.61–0.96), siponimod (ROM = 0.77; 95%-CI: 0.60–0.98), and fingolimod (ROM = 0.83; 95%-CI: 0.71–0.96). The treatment effect on BVL was associated with the treatment effect on disability accumulation (β = 0.466; p = 0.008), and this association remained significant independently of the treatment effect on MRI activity (β = 0.422; p = 0.005).

**Interpretation:**

Several DMTs—including newer therapies—significantly reduce BVL, and this effect correlates with reduced disability accumulation. These findings support BVL as a meaningful treatment target in MS.

**Funding:**

None.

**Research in context**

**Evidence before this study**

Multiple sclerosis (MS) involves both inflammatory and neurodegenerative mechanisms, with brain volume loss (BVL) serving as a key marker of neurodegeneration and a predictor of long-term disability. Although many disease-modifying therapies (DMTs) have demonstrated the ability to reduce BVL, direct comparisons of their relative effects remain limited, partly due to the scarcity of head-to-head randomized trials. Earlier studies used placebo controls, while more recent trials rely on active comparators, complicating cross-study comparisons. A meta-analysis from 2014 identified an association between treatment effects on BVL and disability progression but did not include several DMTs approved in subsequent years. As a result, a comprehensive, up-to-date synthesis of the comparative impact of all approved DMTs on BVL—and how these effects relate to disability accumulation—is lacking. We searched PubMed, Embase, and Cochrane from inception to September 2024, without language restrictions, using terms for multiple sclerosis (“Multiple Sclerosis”, “MS”), trial design (“Randomized Controlled Trial”, “RCT”), and the following DMTs: alemtuzumab, cladribine, dimethyl fumarate, diroximel fumarate, fingolimod, glatiramer acetate, interferon beta-1a, interferon beta-1b, natalizumab, ocrelizumab, ofatumumab, ozanimod, peg-interferon beta-1a, ponesimod, siponimod, teriflunomide, and ublituximab. Eligible trials enrolled adults, assessed treatment effects on BVL over ≥1 year, and excluded pediatric populations, combination therapies, and open-label or extension studies.

**Added value of this study**

This study provides a thorough network meta-analysis of 33 randomized controlled trials encompassing over 26,000 patients, systematically evaluating the efficacy of 16 approved DMTs on BVL, MRI lesion activity, and disability progression. Eight therapies showed significant superiority over placebo in reducing BVL, while all reduced MRI lesion accumulation, and six significantly delayed disability worsening. Meta-regression analyses revealed that reductions in BVL were associated with decreased disability accumulation, independent of changes in MRI lesion activity. By integrating both direct and indirect evidence, this work advances prior knowledge by robustly establishing BVL reduction as an independent and clinically meaningful predictor of long-term disability outcomes across a broad spectrum of modern treatments.

**Implications of all the available evidence**

These findings underscore that multiple DMTs—including newer agents—effectively reduce BVL in MS. The demonstrated link between treatment effects on BVL and disability accumulation, independent of inflammatory lesion activity, suggests that some therapies may provide neuroprotective benefits beyond MRI lesion control. Furthermore, these results strengthen the role of BVL as a clinically relevant biomarker and a meaningful therapeutic target in MS management.

## Introduction

Multiple sclerosis (MS) is a chronic, immune-mediated disease of the central nervous system (CNS), characterized by the coexistence of inflammatory and neurodegenerative processes.^1^ While a wide range of disease-modifying therapies (DMTs) is now available, these treatments primarily target the inflammatory component, especially in patients with relapsing-remitting MS. In contrast, the neurodegenerative component—recognized as the primary driver of long-term disability^2^—remains less effectively addressed.^1^

Inflammatory activity in MS manifests clinically as relapses and radiologically as the accumulation of focal lesions. Neurodegeneration, by contrast, can be quantified through brain volume loss (BVL), a process that occurs at a pathologically accelerated rate in MS.^3^ Notably, increased BVL is consistently observed across all disease phases, including its early stages.^4^ At the population level, BVL correlates strongly with greater disability severity and is a predictor of future disease progression, associated with both physical and cognitive impairment.^4^, ^5^, ^6^, ^7^ Beyond its prognostic value, BVL offers critical insights into the mechanisms driving progression and has been proposed as a key outcome measure in clinical trials investigating neuroprotective therapies.^4^

Although several DMTs have demonstrated efficacy in reducing BVL in MS,^8^ systematic comparisons of their effects remain challenging due to the limited availability of head-to-head trials. Evolution in trial design, with older clinical trials primarily assessing treatment effects against placebo and more recent studies using active comparators, further complicates the interpretation of relative treatment effects.

Furthermore, the extent to which reductions in BVL correlate with improvements in disease trajectories in terms of disability progression, remains incompletely understood. A meta-analysis published in 2014 established a link between treatment effects on BVL and disability accumulation.^9^ However, the approval of several new DMTs since then necessitates an updated and comprehensive analysis. Such an effort could not only further validate BVL as a clinically meaningful measure of disease progression but also enhance our understanding of how treatment-induced reductions in BVL influence long-term outcomes.

To address these gaps, we conducted a study with two complementary components: 1) a network meta-analysis (NMA), a statistical approach that integrates direct evidence from head-to-head trials and indirect evidence from trials with a common comparator, to systematically compare the effects of DMTs on BVL. This method enables the estimation of relative treatment effects even when direct comparisons between treatments are unavailable^10^; 2) a meta-regression, designed to investigate the association between treatment effects on BVL and disability accumulation. Meta-regression is a statistical approach that enables the exploration of relationships between outcomes of study-level characteristics across trials.

## Methods

The study adhered to the Preferred Reporting Items for Systematic Reviews and Meta-analyses (PRISMA) guidelines for NMAs.^11^

### Search strategy and selection criteria

We included randomized controlled trials (RCTs) on adult patients with MS reporting treatment effect on BVL over a period of at least one year for any of the following FDA-approved DMTs: alemtuzumab, cladribine, dimethyl fumarate, diroximel fumarate, fingolimod, glatiramer acetate, interferon beta-1a, interferon beta-1b, natalizumab, ocrelizumab, ofatumumab, ozanimod, peg-interferon beta-1a, ponesimod, siponimod, teriflunomide, and ublituximab. RCTs involving pediatric populations, DMT combinations, or open-label or extension phases were excluded.

**Fig. 1:**
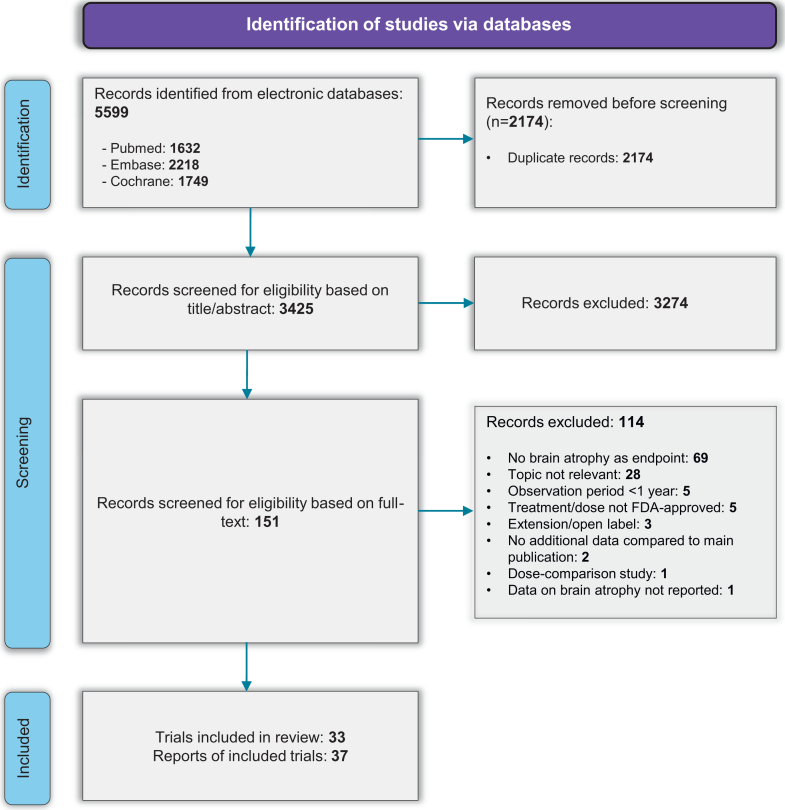
**PRISMA flow diagram**.

A comprehensive search of PubMed, Embase, and the Cochrane Registry of Clinical Trials was conducted from database inception to September 2024, without language restrictions. Detailed search strategies are provided in eMethods, and the screening process is summarized in the PRISMA flow diagram in Fig. 1.

### Data analysis

Two reviewers (A.C. and R.P.) independently extracted data, resolving discrepancies by consensus. For each trial, we recorded publication year, number of randomized patients, and key demographic and clinical characteristics. Outcomes of interest included treatment effects on BVL, MRI lesions, and probability of disability progression. These were evaluated as the ratio of mean (ROM) or median brain volume change, the ratio of cumulative active T2-lesions counts, and the ratio of disability progression probability between the intervention and control arms.^9^ BVL estimates excluding the first 6 (or 12) months of observation were used to account for pseudoatrophy effects where available. Further details are provided in eMethods.

All statistical analyses were conducted using *R* (version 4.2.1; R Core Team, 2022), with a significance threshold set at p < 0.05. Treatment effects on the outcomes of interest were analyzed using NMAs with the *netmeta* package,^12^ employing mixed-effects models with placebo as the reference group. Risk of bias was assessed systematically (eTable 1), model inconsistency was evaluated by comparing indirect effects with direct effects where available (eFigures 1–3), and publication bias was assessed with funnel plots and Egger's test (eFigures 4–6). Sensitivity analyses included: 1) Bayesian NMA models with evaluation of treatment rankings using surface under the cumulative ranking (SUCRA) values. SUCRA provides a summary score for each treatment, indicating the probability of being among the most effective options, with values closer to 100% reflecting better performance; 2) analyses restricted to patients with relapsing MS (RMS); 3) analyses restricted to BVL estimates obtained with Structural Image Evaluation using Normalization of Atrophy (SIENA) software^13^; 4) analyses using the longest BVL observation period; 5) analyses limited to studies excluding the initial 6 (or 12) months for BVL estimation; 6) exclusion of RCTs in which measures of uncertainty could only be imputed; and 7) analyses restricted to RCTs reporting disability accumulation confirmed at 3 months.

Meta-regression analyses were conducted using linear regression models to assess the association between the treatment effect on BVL and the treatment effect on disability progression. The association was explored both in a univariable analysis and including the treatment effect on MRI lesions as an additional covariate. Each trial was given a weight based on the number of participants and the length of the observation period, as previously proposed.^9^ The coefficient of determination (R^2^) was used to assess the goodness of fit for each model. Models were applied using both the treatment effects derived from the original RCTs, as well as the estimates derived from the NMAs. Sensitivity analyses were conducted: 1) excluding each individual trial, 2) using unweighted regression, 3) in patients with RMS only and in patients with RRMS only, 4) using BVL estimate referred to the longest observation period, 5) in models adjusted for the software used to estimate BVL, and 6) in models accounting for cohort age, sex, and expanded disability status scale (EDSS) score (eTables 2–5). In addition, we performed a causal mediation analysis to explore the extent to which the association between treatment effects on BVL and disability progression was mediated by changes in MRI lesion accumulation. The analysis was implemented using the *mediation* R package with nonparametric bootstrapping (1000 simulations).

### Role of the funding source

There was no funding source for this study.

## Results

The systematic literature review identified 37 eligible studies for data extraction, including primary RCT publications and post-hoc analyses. These studies were derived from 33 unique RCTs evaluating 16 different DMTs and included a total of 26,247 patients with MS: 21,309 with RMS, and 4938 with progressive MS (PMS). When multiple publications originated from the same RCT, we included only non-overlapping data for each outcome of interest to avoid duplication of participant populations. All steps of the study selection process are detailed in the PRISMA flow diagram shown in Fig. 1. Among studies on RMS, the weighted mean (SD) age was 36.8 (1.6) years, the proportion of female participants was 68.4% (3.4), and the mean EDSS was 2.6 (0.2). In studies on PMS, the weighted mean (SD) age was 46.6 (2.6) years, the proportion of female participants was 56.7% (6.2), and the mean EDSS was 5.2 (0.4). Additional details are reported in Table 1 and eTable 6.

**Table 1:** Characteristics of included trials.

Trial	Experimental arm	N experimental arm	Control arm	N control arm	Mean age experimental arm	% Females experimental arm	Mean EDSS experimental arm	MS phenotype
MSCRG (1996)^14^^,^^15^	Interferon β-1a	158	Placebo	143	36.7	75	2.4	RRMS
IFNβ-1b European Study Group (1998)^16^^,^^17^	Interferon β-1b	360	Placebo	358	41.1	58	5.1	SPMS
AFFIRM (2006)^18^^,^^19^	Natalizumab	627	Placebo	315	35.6	72	2.3	RRMS
REGARD (2008)^20^	Interferon β-1a	386	Glatiramer acetate	378	36.7	69	2.4	RRMS
CAMMS223 (2008)^21^	Alemtuzumab	112	Interferon β-1a	111	31.9	64	1.9	RRMS
BEYOND (2009)^22^	Interferon β-1b	897	Glatiramer acetate	448	35.8	70	2.4	RRMS
Montalban et al. (2009)^23^	Interferon β-1b	36	Placebo	37	48.8	39	5.3	PPMS
FREEDOMS I (2010)^24^	Fingolimod	425	Placebo	418	36.6	70	2.3	RRMS
TRANSFORMS (2010)^25^	Fingolimod	431	Interferon β-1a	435	36.7	65	2.2	RRMS
TEMSO (2011)^26^^,^^27^	Teriflunomide	359	Placebo	363	37.8	71	2.7	RRMS
CONFIRM (2012)^28^^,^^29^	Dimethyl fumarate	359	Placebo	363	37.8	68	2.6	RRMS
	Glatiramer acetate	350			36.7	71	2.6	RRMS
CARE-MS I (2012)^30^	Alemtuzumab	376	Interferon β-1a	187	33.0	65	2.0	RRMS
CARE-MS II (2012)^31^	Alemtuzumab	426	Interferon β-1a	202	34.8	66	2.7	RRMS
GALA (2013)^32^	Glatiramer acetate	943	Placebo	461	37.4	68	2.8	RRMS
DEFINE (2014)^33^^,^^34^	Dimethyl fumarate	410	Placebo	408	38.1	72	2.4	RRMS
ADVANCE (2014)^35^	Peginterferon β-1a	512	Placebo	500	36.9	71	2.5	RRMS
FREEDOMS II (2014)^36^	Fingolimod	358	Placebo	355	40.6	77	2.4	RRMS
INFORMS (2016)^37^	Fingolimod	336	Placebo	487	49.0	49	4.7	PPMS
OPERA I (2017)^38^	Ocrelizumab	410	Interferon β-1a	411	37.1	66	2.9	RRMS
OPERA II (2017)^38^	Ocrelizumab	417	Interferon β-1a	418	37.2	65	2.8	RRMS
ORATORIO (2017)^39^	Ocrelizumab	488	Placebo	244	44.7	49	4.7	PPMS
CLARITY (2018)^40^^,^^41^	Cladribine	433	Placebo	437	37.9	69	2.8	RRMS
EXPAND (2018)^42^	Siponimod	1105	Placebo	546	48.0	61	5.4	SPMS
ASCEND (2018)^43^	Natalizumab	439	Placebo	448	47.3	62	6.0^a^	SPMS
SUNBEAM (2019)^44^	Ozanimod	447	Interferon β-1a	448	34.8	63	2.6	RMS
RADIANCE (2019)^45^	Ozanimod	433	Interferon β-1a	441	36.0	67	2.6	RMS
ASCLEPIOS I (2020)^46^	Ofatumumab	465	Teriflunomide	462	38.9	68	3.0	RMS
ASCLEPIOS II (2020)^46^	Ofatumumab	481	Teriflunomide	474	38.0	66	2.9	RMS
ASSESS (2020)^47^	Fingolimod	352	Glatiramer acetate	342	40.3	75	2.7	RRMS
OPTIMUM (2021)^48^	Ponesimod	567	Teriflunomide	566	36.7	64	2.6	RRMS
FUMAPMS (2021)^49^	Dimethyl fumarate	27	Placebo	27	55.7	37	4.3	PPMS
ULTIMATE I (2022)^50^	Ublituximab	271	Teriflunomide	274	36.2	61	3.0	RMS
ULTIMATE II (2022)^50^	Ublituximab	272	Teriflunomide	272	34.5	65	2.8	RMS
*Total*								

Abbreviations: RMS, relapsing multiple sclerosis; RRMS, relapsing-remitting multiple sclerosis; PPMS, primary-progressive multiple sclerosis; SPMS, secondary-progressive multiple sclerosis.

a Median.

### Treatment effect on BVL

**Fig. 2:**
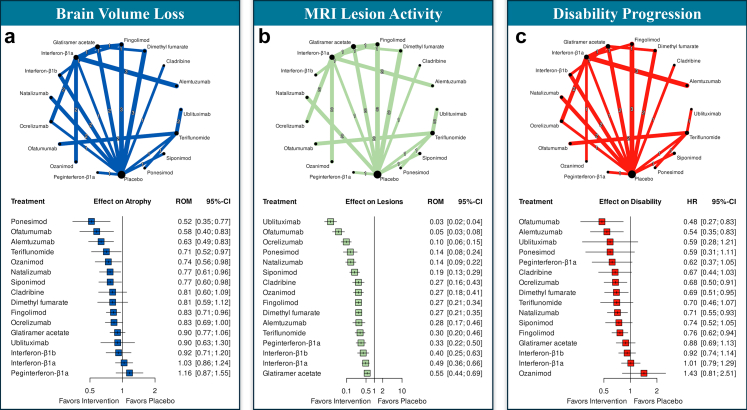
**Results of the network meta-analyses**. a: treatment effect on brain volume loss; b: treatment effect on MRI lesion activity; c: treatment effect on disability progression. Each panel includes two elements: 1) Top: A network diagram summarizing direct comparisons between disease-modifying treatments across randomized controlled trials. Each node represents a treatment, with node size proportional to the total number of participants receiving that treatment. Edges indicate direct comparisons, with thickness reflecting the number of contributing trials, and the exact number of trials labeled at the midpoint of each edge; 2) Bottom: Forest plots displaying treatment effects estimated from NMA models, compared to placebo. For brain volume loss and MRI lesion activity (Panels a and b), results are reported as ratios of means (ROM); for disability progression (Panel c), effects are reported as hazard ratios (HR). Squares represent point estimates, and horizontal lines represent 95% confidence intervals. Results of the corresponding sensitivity analyses are presented with analogous network diagrams and forest plots in eFigures 8–15. Abbreviations: ROM, ratio of means; HR, hazard ratio.

All included RCTs reported data on BVL, as per inclusion criteria. The median (IQR) observation time was 24 (18–24) months. BVL was estimated using SIENA in 18 RCTs, with other or unspecified software used in the remaining 15. Results of the NMA are reported in Fig. 2a. Eight DMTs showed significant superiority over placebo in reducing BVL, including ponesimod (ROM = 0.52; 95%-CI: 0.35–0.77), ofatumumab (ROM = 0.58; 95%-CI: 0.40–0.83), alemtuzumab (ROM = 0.63; 95%-CI: 0.49–0.83), teriflunomide (ROM = 0.71; 95%-CI: 0.52–0.97), ozanimod (ROM = 0.74; 95%-CI: 0.56–0.98), natalizumab (ROM = 0.77; 95%-CI: 0.61–0.96), siponimod (ROM = 0.77; 95%-CI: 0.60–0.98), and fingolimod (ROM = 0.83; 95%-CI: 0.71–0.96). Bayesian NMA yielded similar results (eFigure 7).

In the sensitivity analysis estimating BVL based on the longest observation period, six DMTs were superior to placebo in reducing BVL, including ponesimod (ROM = 0.52; 95%-CI: 0.35–0.77), ofatumumab (ROM = 0.58; 95%-CI: 0.40–0.83), alemtuzumab (ROM = 0.64; 95%-CI: 0.49–0.83), teriflunomide (ROM = 0.71; 95%-CI: 0.52–0.97), ozanimod (ROM = 0.75; 95%-CI: 0.57–0.98), and fingolimod (ROM = 0.82; 95%-CI: 0.71–0.95) (eFigure 8). Results of sensitivity analyses are reported in eFigures 8–15. In sensitivity analyses, results were largely consistent with those of the main analyses. Among DMTs tested in the RMS population, as well as in purely RRMS population, those showing significant effect on BVL in the main analyses were confirmed in these subgroups. When the NMA was restricted to RCTs reporting BVL estimates with rebaselining, ocrelizumab also demonstrated a significant effect on BVL. Conversely, when longer observation periods were prioritized over the rebaselining time frame, the primary change was a loss of significance for natalizumab. Results were consistent when excluding RCTs in which measures of uncertainty had to be imputed.

### Treatment effect on MRI lesion activity

MRI lesion activity was measured by the count of active T2-lesions (i.e., new or enlarging T2-lesions) in 27 RCTs, and by the count of combined unique active lesions (i.e., new or enlarging T2-lesions or gadolinium-enhancing lesions) in 2 studies. The median (IQR) observation time was 24 (24–24) months. In the NMA, all DMTs demonstrated significant superiority over placebo in preventing MRI lesion activity, with ROM ranging from 0.03 to 0.55 (Fig. 2b). Bayesian NMA yielded similar results (eFigure 16).

### Treatment effect on disability accumulation

Data on confirmed disability progression were available for 31 RCTs, with hazard ratio (HR) reported in 23 RCTs and risk ratio (RR) in 8. The median (IQR) observation time was 24 (24–24) months. In the NMA, six DMTs showed significant superiority over placebo in preventing disability accumulation, including ofatumumab (HR = 0.48; 95%-CI: 0.27–0.83), alemtuzumab (HR = 0.54; 95%-CI: 0.35–0.83), ocrelizumab (HR = 0.68; 95%-CI: 0.50–0.91), dimethyl fumarate (HR = 0.69; 95%-CI: 0.51–0.95), natalizumab (HR = 0.71; 95%-CI: 0.55–0.93), and fingolimod (HR = 0.76; 95%-CI: 0.62–0.94) (Fig. 2c). Bayesian NMA yielded similar results (eFigure 17).

### Association between treatment effects on BVL and disability

**Fig. 3:**
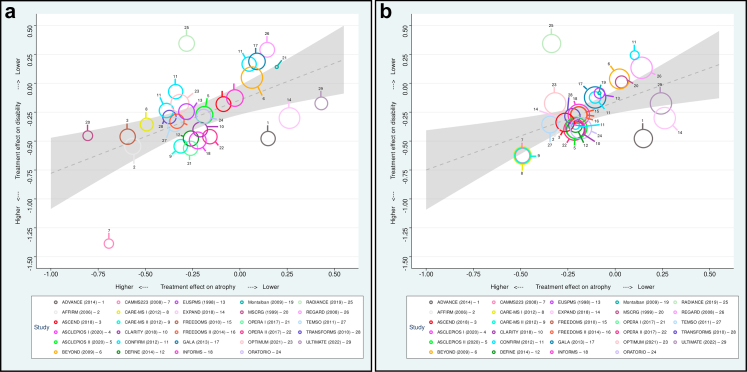
**Association between treatments effects on brain volume loss and disability accumulation**. Panel a: Weighted regression model using treatment estimates derived from the original RCTs. Panel b: Weighted regression model using treatment estimates derived from the NMAs. The circle size reflects the weight of the study in the weighted regression.

In the weighted meta-regression, the treatment effect on BVL was significantly associated with the treatment effect on disability progression (β = 0.466; p = 0.008; R^2^ = 0.217) (Fig. 3a). This association remained significant in a multivariable model adjusting for treatment effect on MRI lesion activity (β = 0.422; p = 0.005; R^2^ = 0.560) with minimal change in the strength of association between treatment effect on BVL and disability when adjusting for treatment effect on MRI lesions (β decreased from 0.466 to 0.422, indicating that less than 10% of the relationship between treatment effect on BVL and disability is mediated by the effect on MRI lesions). Adjustment for treatment effect on MRI lesions had a relevant effect on the explained variance (R^2^ increased from 0.217 to 0.560, indicating a complementary and independent effect of MRI lesions on disability effect). Similar results were obtained when using treatment effect estimates derived from the NMA models (univariable regression: β = 0.582; p = 0.0006; R^2^ = 0.339; multivariable regression accounting for MRI lesion activity: β = 0.392; p = 0.012; R^2^ = 0.549) (Fig. 3b). Results were confirmed in sensitivity analyses (eTables 2–5). The variance in treatment effect on disability progression explained by the treatment effect on BVL was increased in sensitivity analyses restricted to specific subsets of RCTs: RCTs conducted in RRMS populations (R^2^ = 0.435), those using SIENA or brain parenchymal fraction (BPF) for BVL estimation (R^2^ = 0.431), and those reporting BVL with rebaselining (R^2^ = 0.503) (eTable 3). A causal mediation analysis indicated that the association between treatment effects on BVL and disability accumulation was not significantly mediated by MRI lesion accumulation (eTable 7). For all three outcomes of interest, assessment of funnel plot asymmetry and Egger's test revealed no substantial evidence of publication bias (eFigures 4–6).

## Discussion

Using a meta-analytical approach, we showed that multiple DMTs significantly reduce the rate of BVL in patients with MS compared to placebo. Additionally, multiple treatments decreased the risk of confirmed disability progression, while all approved DMTs effectively reduced the accumulation of MRI lesions, a key marker of focal inflammatory activity. Notably, the reduction in BVL was associated with decreased disability accumulation, with this relationship being independent of the treatments' effects on MRI lesions. These findings suggest that certain DMTs may impact neurodegenerative processes in MS beyond their impact on neuroinflammation.

Among the 16 DMTs analyzed, eight demonstrated a significant effect in reducing BVL. These included sphingosine 1-phosphate (S1P) modulators (ponesimod, ozanimod, siponimod, fingolimod), monoclonal antibodies (ofatumumab, alemtuzumab, natalizumab), and teriflunomide. Additionally, ocrelizumab showed a trend toward statistical significance. Notably, more recently approved DMTs were generally more effective in mitigating BVL, reflecting advancements in therapeutic development. Among these, ponesimod exhibited the greatest efficacy, nearly halving the rate of atrophy compared to placebo.

Six DMTs also significantly reduced the risk of disability accumulation compared to placebo, including monoclonal antibodies (ofatumumab, alemtuzumab, ocrelizumab, natalizumab), S1P modulators (fingolimod), and dimethyl fumarate. Ofatumumab exhibited the most substantial effect, lowering disability accumulation risk by over 50%. As expected, all DMTs effectively decreased MRI lesion accumulation, with reductions ranging from 45% for glatiramer acetate to an almost complete suppression (97%) observed with ublituximab. Bayesian analyses yielded largely concordant results, with the added advantage of providing SUCRA values—a summary measure that reflects the probability (expressed as a percentage) that a treatment ranks among the most effective in the network, thereby supporting its potential value in treatment decision-making. Notably, the observed effects of DMTs on BVL remained largely consistent across sensitivity analyses. Analyses restricted to relapsing populations confirmed the significant impact on BVL of DMTs identified in the main analysis. Results were also consistent when considering different time frames for BVL estimation. The main difference was the loss of a significant effect for natalizumab when prioritizing longer observation periods over the rebaselining time frame, likely reflecting the influence of pseudoatrophy. Importantly, natalizumab is known to produce a pronounced pseudoatrophy effect.^4^^,^^18^ Conversely, when restricting the analysis to trials without rebaselining, ocrelizumab emerged as having a significant effect on BVL, suggesting a true treatment effect that may have been obscured in more heterogeneous trial settings.

These findings were derived using a network meta-analytic framework that integrates both direct and indirect comparisons across approved DMTs. While multiple head-to-head RCTs were available, they covered only a limited subset of treatment pairs: specifically, 22 unique direct comparisons for BVL, 20 for MRI lesion accumulation, and 21 for disability progression. The majority of the possible pairwise comparisons (n = 136) for each outcome were therefore informed indirectly through the network structure. In this context, NMA enabled a unified assessment of relative treatment effects across all outcomes, extending beyond the capabilities of conventional pairwise meta-analyses.

Meta-regression analyses showed a positive association between the treatment effects on BVL and disability progression, even after accounting for the treatment effects on MRI activity, a known contributor to brain atrophy. This analysis is in accordance with one earlier meta-analysis^9^ reporting that the treatment effect on BVL influences disability progression independently and complements the treatment effect on MRI lesions. This suggests that certain DMTs may exert effects on neurodegenerative processes in MS independent of their effects of acute inflammation. This consideration is particularly relevant given the growing recognition of progression independent of relapse activity (PIRA) as a major driver of disability accumulation in MS,^51^^,^^52^ even in patients with typical relapsing-remitting phenotype.^51^^,^^53^^,^^54^ Remarkably, recent studies have shown that patients with PIRA still exhibit accelerated BVL,^55^^,^^56^ even when both clinical and MRI inflammatory activity are completely suppressed.^55^ Although PIRA has not yet been included as an outcome measure in MS trials, DMT use has been shown to delay reaching disability milestones in patients experiencing PIRA.^52^ Additionally, post-hoc analyses of ocrelizumab, ofatumumab, and ponesimod trials have supported a positive impact of these treatments in reducing the incidence of PIRA.^51^^,^^53^^,^^57^

The impact of treatment effect on neurodegeneration, along with the reduced risk of relapse-associated worsening, may explain the observed improvement in clinical trajectories in the treatment era compared to earlier periods. In fact, several observational studies have shown that the rates of disability accrual and progression to secondary progressive MS are now substantially lower compared to previous natural history studies.^58^ Additionally, early initiation of high-efficacy therapies is especially linked to better long-term outcomes.^59^

Remarkably, our study found that the treatment effect on BVL accounted for nearly 22% (R^2^ = 0.217) of the variability in the effect on disease progression, highlighting the potential involvement of other biological mechanisms. Factors such as non-resolving inflammation in chronic active lesions,^60^, ^61^, ^62^ leptomeningeal inflammation,^63^ cortical lesions,^64^^,^^65^ diffuse microglial activation, chronic demyelination, oxidative stress, mitochondrial dysfunction, and the failure of compensatory mechanisms^66^ have all been proposed as contributors to disease progression. Importantly, these mechanisms may not always manifest with measurable atrophy. Additionally, spinal cord atrophy plays a pivotal role in explaining disability progression,^62^^,^^67^^,^^68^ as do regional thalamic and cortical atrophy,^4^^,^^55^^,^^69^, ^70^, ^71^, ^72^ showing a stronger correlation with disease severity than total brain atrophy.^4^ However, their routine quantification in clinical trials remains limited, primarily due to technical challenges.^4^^,^^73^ The heterogeneity of biological processes underlying disability progression but also BVL may explain the dissociation between treatment effects on BVL and disability progression seen with certain treatments, as recently observed with the Bruton tyrosine kinase (BTK) inhibitor tolebrutinib.^74^^,^^75^

The proportion of variance in treatment effect on disability accumulation explained by BVL was lower than previously reported by Sormani et al.^9^ However, that study applied more restrictive inclusion criteria, focusing on RCTs conducted in RRMS populations, lasting at least 24 months, using SIENA or BPF for BVL estimation, and including rebaselined BVL measures. When similar criteria were applied in our sensitivity analyses, the explanatory power of BVL increased substantially, reaching up to 70% (R^2^ = 0.703).

The results of this study should be interpreted with caution, as NMA relies on several critical assumptions that may not have been fully satisfied in this context. These include homogeneity (expecting similar results across studies comparing the same treatments), transitivity (ensuring comparability of treatment effect across studies), consistency (agreement between direct and indirect evidence), and similarity across study designs, populations, and outcomes. Since our analysis prioritized maximizing data inclusion to provide a comprehensive overview of treatment effects, this approach may have increased the risk of bias that we tried to address with some of the sensitivity analyses.

Several potential sources of bias and violations of these assumptions warrant careful consideration. First, technical variability in MRI acquisition protocols and sequence parameters may have introduced inconsistencies. Additionally, different post-processing methods were used to estimate BVL: while most RCTs employed SIENA, others used alternative tools with varying sensitivity to detect neurodegenerative changes and differing susceptibility to measurement error.^76^

Second, outcome definitions and reporting practices varied across studies. For BVL, some studies reported annualized percentage change, while others presented absolute change over differing time frames. Definitions of MRI lesion accumulation also differed, with some studies including only new or enlarging T2 lesions and others incorporating gadolinium-enhancing lesions. For disability accumulation, outcome measures ranged from time-to-event analyses to binary outcomes reported as risk ratios, complicating direct comparisons.

Third, study duration varied widely, which may have influenced the ability to detect treatment effects and introduced variability in outcome sensitivity. In some cases, time frames differed across outcomes within the same study, further limiting comparability.

Fourth, differences in study populations may have challenged the transitivity assumption. Inclusion criteria varied in terms of disease duration, baseline EDSS, prior treatment exposure—including RCTs restricted to treatment-naive patients—and pre-enrollment relapse rates. Additionally, we intentionally included RCTs involving both relapsing and progressive MS populations. Although BVL rates have been reported as similar between these phenotypes,^4^ their inclusion may still affect transitivity.

Fifth, due to data limitations, we were unable to systematically exclude the initial observation period in all trials when quantifying BVL, potentially introducing bias related to the pseudoatrophy effect.^4^^,^^73^

Sixth, studies differed in their statistical methodologies, with some reporting adjusted treatment effects and others providing unadjusted outcomes, limiting direct comparability.

Finally, although we formally tested for publication bias—including enough studies to allow such assessment—and found no significant evidence of its presence, its potential influence cannot be entirely ruled out, as is common in meta-analysis.

While extensive sensitivity analyses were conducted to address these limitations and evaluate the robustness of our findings, residual bias may remain. These concerns are particularly relevant given the limited number of direct comparisons available for each outcome, meaning that many estimates relied primarily on indirect evidence. However, in cases where both direct and indirect evidence were available, the estimates derived from the NMA were largely consistent with those from head-to-head RCTs, providing reassurance regarding the internal coherence of the model.^17^^,^^73^^,^^76^ Another important limitation lies in the inherent observational nature of NMA. As such, it cannot establish causality and does not replace direct head-to-head comparisons, which remain the gold standard for evaluating treatment efficacy.

It is also important to emphasize that the associations observed in the meta-regression analyses are based on group-level data, not individual-level data. These associations do not establish causality but indicate that the effects of treatment on the studied outcomes occur concurrently. As such, they are subject to ecological bias, whereby group-level associations may not reflect true relationships at the individual level. Moreover, meta-regression is inherently limited by the number of available studies, resulting in reduced statistical power and the ability to detect only large associations. Additionally, most studies included in this work had a follow-up of 24 months or less, limiting the ability to capture long-term disability accumulation and neurodegeneration. This constrains the interpretation of the BVL–disability accumulation relationship, particularly for DMTs whose effects may evolve over time. As a result, potential changes in long-term effects on both BVL and disability accumulation may be underestimated.

From a methodological perspective, the use of ROMs as the effect size metric assumes an underlying log-normal distribution of outcomes, which may not always hold. Moreover, while ROMs offer advantages in terms of comparability across trials, their interpretability is less intuitive than that of more commonly used metrics, such as standardized mean difference.

Despite these limitations, this study offers valuable insights by demonstrating that several DMTs substantially reduce BVL in MS, with this being linked to reduced disability progression. These findings collectively highlight the importance of assessing BVL in trials targeting long-term disability accrual, reinforcing its significance as a meaningful therapeutic target.

## Contributors

AC drafted the manuscript. AC, SS, LK, MPS, CG contributed to the study design and methodology. AC, RP conducted the literature search, data extraction, and synthesis of results. AC and SS conducted the statistical analysis. All authors contributed to critical revision of the manuscript for important intellectual content and gave final approval for the decision to submit for publication. AC, RP, and SS accessed and verified the data. All authors had full access to all data included in the study and take final responsibility for the decision to submit for publication.

## Data sharing statement

Data in this systematic review and meta-analysis were extracted from published studies available elsewhere.

## Declaration of interests

AC received speaker honoraria from Novartis and Roche. SS has nothing to disclose. RP is supported by the Gottfried & Julia Bangerter–Rhyner Foundation and the swiss academies of arts and sciences, received funding from the Hertie foundation and travel funds from Teva and Roche. LK has received no personal compensation. His institutions (University Hospital Basel/Foundation Clinical Neuroimmunology and Neuroscience Basel) have received and used exclusively for research support: payments for steering committee and advisory board participation, consultancy services, and participation in educational activities from: Actelion, Bayer, BMS, df-mp Molnia & Pohlmann, Celgene, Eli Lilly, EMD Serono, Genentech, Glaxo Smith Kline, Janssen, Japan Tobacco, Merck, MH Consulting, Minoryx, Novartis, F. Hoffmann-La Roche Ltd, Senda Biosciences Inc., Sanofi, Santhera, Shionogi BV, TG Therapeutics, and Wellmera, and license fees for Neurostatus-UHB products; grants from Novartis, Innosuisse, and Roche. MPS received consulting fees from Biogen, Merck, Novartis, Roche, Sanofi, Immunic, Alexion. CG: The University Hospital Basel (USB) and the Research Center for Clinical neuroimmunology and Neuroscience (RC2NB), as the employers of Cristina Granziera, have received the following fees which were used exclusively for research support from Siemens, GeNeuro, Genzyme-Sanofi, Biogen, Roche. They also have received advisory board and consultancy fees from Actelion, Genzyme-Sanofi, Novartis, GeNeuro, Merck, Biogen and Roche; as well as speaker fees from Genzyme-Sanofi, Novartis, GeNeuro, Merck, Biogen, and Roche.

## Appendix A Supplementary data

eMaterials
