# Supplementary material for: The effect of disease-modifying therapies on brain volume loss and disability accumulation in multiple sclerosis: a systematic review and network meta-analysis
Source: Lancet Reg Health Eur. 2025 Sep 27;59:101476. doi: 10.1016/j.lanepe.2025.101476 (PMC12509907; doi:10.1016/j.lanepe.2025.101476)
Supplement: eMaterials [file mmc1.docx]

**Supplemental Online Content**

**eMethods**

**eTable 1. Quality Rating Scheme for the Included Studies**

**eTable 2. Sensitivity Analyses on the Association Between Treatment Effects on Brain Volume Loss and Disability Progression**

**eTable 3. Sensitivity Analyses on the Association Between Treatment Effects on Brain Volume Loss and Disability – Univariate Associations**

**eTable 4. Meta-regression: Association between treatment effect on BVL and treatment effect on disability accumulation (with 3-month confirmation), accounting for treatment effect on MRI lesions – Weighted regression model using treatment estimates derived from original RCTs**

**eTable 5. Meta-regression: Association between treatment effect on BVL and treatment effect on disability accumulation (with 3-month confirmation), accounting for treatment effect on MRI lesions – Weighted regression model using treatment estimates from the NMAs**

**eTable 6. Characteristics of RCTs reporting BVL Estimates**

***eTable 7. Causal mediation analysis – Mediation of the relationship between treatment effect on BVL and treatment effect on disability accumulation by treatment effect on MRI lesion accumulation***

**eFigure 1. Comparison of Direct and Indirect Estimates in Network Meta-Analysis for Brain Volume Loss**

**eFigure 2. Comparison of Direct and Indirect Estimates in Network Meta-Analysis for MRI Lesion Activity**

**eFigure 3. Comparison of Direct and Indirect Estimates in Network Meta-Analysis for Disability Progression**

**eFigure 4. Funnel Plot – Brain Volume Loss**

**eFigure 5. Funnel Plot – MRI Lesion Activity**

**eFigure 6. Funnel Plot – Disability Progression**

**eFigure 7. SUCRA Rankings from Bayesian Network Meta-Analysis of Treatment Effect on Brain Volume Loss**

**eFigure 8. Sensitivity Analysis of Network Meta-Analysis Results: Treatment Effect on BVL Using the Longest Observation Period**

**eFigure 9. Sensitivity Analysis of Network Meta-Analysis Results: Treatment Effect on BVL in RCTs conducted in RMS**

**eFigure 10. Sensitivity Analysis of Network Meta-Analysis Results: Treatment Effect on BVL in RCTs using SIENA**

**eFigure 11. Sensitivity Analysis of Network Meta-Analysis Results: Treatment Effect on BVL in RCTs With Rebaseline**

**eFigure 12. Sensitivity Analysis of Network Meta-Analysis Results: Treatment Effect on BVL Excluding RCTs in Which Measures of Uncertainty Could Only Be Imputed**

**eFigure 13. Sensitivity Analysis of Network Meta-Analysis Results: Treatment Effect on MRI Lesion Activity Excluding RCTs in Which Measures of Uncertainty Could Only Be Imputed**

**eFigure 14. Sensitivity Analysis of Network Meta-Analysis Results: Treatment Effect on Disability Progression Prioritizing Measures of Confirmed Disability Progression at Three Months**

**eFigure 15. Sensitivity Analysis of Network Meta-Analysis Results: Treatment Effect on BVL in RCTs conducted in RRMS**

**eFigure 16. SUCRA Rankings from Bayesian Network Meta-Analysis of Treatment Effect on MRI Lesion Activity**

**eFigure 17. SUCRA Rankings from Bayesian Network Meta-Analysis of Treatment Effect on Disability Progression**

**eMethods**

**Search strategy used for the systematic literature review**

The search was conducted on September 24^th^, 2024, as follows:

- Pubmed
  - (("Multiple Sclerosis"[Mesh] OR "Multiple Sclerosis"[Title/Abstract] OR "MS"[Title/Abstract])
    AND ("Alemtuzumab"[Title/Abstract] OR "Interferon"[Title/Abstract] OR "Cladribine"[Title/Abstract] OR "Dimethyl fumarate"[Title/Abstract] OR "BG-12"[Title/Abstract] OR "Diroximel fumarate"[Title/Abstract] OR "Fingolimod"[Title/Abstract] OR "Glatiramer acetate"[Title/Abstract] OR "Haematopoietic stem cell transplantation"[Title/Abstract] OR "HSCT"[Title/Abstract] OR "Natalizumab"[Title/Abstract] OR "Ocrelizumab"[Title/Abstract] OR "Ofatumumab"[Title/Abstract] OR "Ozanimod"[Title/Abstract] OR "peginterferon"[Title/Abstract] OR "Ponesimod"[Title/Abstract] OR "Siponimod"[Title/Abstract] OR "Teriflunomide"[Title/Abstract])
    AND (("Randomized Controlled Trial"[Publication Type] OR "RCT"[Title/Abstract]) OR ("Clinical Trials as Topic"[MeSH])) ).
- Embase:
  - ('multiple sclerosis'/exp OR 'multiple sclerosis':ti,ab OR 'MS':ti,ab)
    AND ('alemtuzumab'/exp OR 'interferon'/exp OR 'cladribine'/exp OR 'dimethyl fumarate'/exp OR 'BG-12':ti,ab OR 'diroximel fumarate':ti,ab OR 'fingolimod'/exp OR 'glatiramer acetate'/exp OR 'haematopoietic stem cell transplantation':ti,ab OR 'HSCT':ti,ab OR 'natalizumab'/exp OR 'ocrelizumab':ti,ab OR 'ofatumumab':ti,ab OR 'ozanimod':ti,ab OR 'peginterferon':ti,ab OR 'ponesimod':ti,ab OR 'siponimod':ti,ab OR 'teriflunomide'/exp)
    AND ('randomized controlled trial'/exp OR 'RCT':ti,ab OR 'clinical trial as topic'/exp).
- Cochrane trials:
  - ("Multiple Sclerosis":ti,ab,kw OR "MS":ti,ab,kw)
    AND ("Alemtuzumab":ti,ab OR "Interferon":ti,ab OR "Cladribine":ti,ab OR "Dimethyl fumarate":ti,ab OR "BG-12":ti,ab OR "Diroximel fumarate":ti,ab OR "Fingolimod":ti,ab OR "Glatiramer acetate":ti,ab OR "Haematopoietic stem cell transplantation":ti,ab OR "HSCT":ti,ab OR "Natalizumab":ti,ab OR "Ocrelizumab":ti,ab OR "Ofatumumab":ti,ab OR "Ozanimod":ti,ab OR "peginterferon":ti,ab OR "Ponesimod":ti,ab OR "Siponimod":ti,ab OR "Teriflunomide":ti,ab) AND ("Randomized Controlled Trial":pt OR "RCT":ti,ab OR "Clinical Trials as Topic":ti,ab,kw).

**Network meta-analysis**

For brain volume loss (BVL), we prioritized time windows that excluded the initial observation period, selecting 6 months (or 12 months if 6 months was unavailable) to minimize the potential pseudoatrophy effect. Pseudo-atrophy refers to the early, treatment-related reduction in brain volume that reflects resolution of inflammation rather than true neurodegeneration and can confound the interpretation of BVL as a marker of disease progression. Specifically, if rebaselining was performed in the original study, we selected BVL values derived from the rebaselined time frame. In cases where rebaselining was performed at both 6 and 12 months, we prioritized the interval starting at 6 months. Among all eligible time windows meeting these criteria, we selected the longest available follow-up duration (e.g., 6–24 months rather than 6–12 months) to maximize sensitivity to treatment effects. In the case of the TEMSO study,^1^ where two methods for estimating BVL were reported, we used the SIENA-derived estimate.^2^

For MRI lesions, when studies reported multiple outcome measures, we prioritized them in the following order: new-enlarging T2 lesions, new T2 lesions, and combined unique active lesions, while excluding studies that reported only T2-lesion volume change^3–6^. This decision was based on the fact that T2-lesion volume change reflects a different construct, capturing not only new lesion accumulation but also fluctuations in the size of chronic lesions, including volume reductions. Unlike new lesion counts, which are unidirectional markers of acute inflammation, lesion volume can decrease over time, making it less comparable to other measures of lesion activity.

For disability progression, we used the occurrence of confirmed disability progression at 6 months as the primary outcome measure, whenever available. If this was not reported, we used confirmed disability progression at 3 months. Studies that did not provide data on confirmed disability progression or only reported changes in EDSS during the observation period were excluded^7,8^. Disability progression outcomes were only pooled for the RADIANCE and SUNBEAM trials,^9,10^ as well as for the ULTIMATE I and ULTIMATE II trials, as reported in the related publications.^11^ In these cases, the pooled estimates were used in the analyses.

To estimate the standard error of the treatment effect (seTE) for network meta-analyses (NMAs), we used the standard deviations of treatment effects reported in the original publications. When standard deviations were not directly reported, they were derived from confidence intervals, interquartile ranges, or standard errors, depending on the available data, assuming normality. In cases where no measures of atrophy variability were reported (for 2 RCTs on BVL and 2 RCTs on MRI lesions), missing standard deviations were imputed using predictive mean matching based on observation duration, participant numbers, and mean atrophy values, employing the *mice* package in *R*.^12^

Bayesian network meta-analyses were conducted using random-effects models with the *gemtc* package in *R*. Markov Chain Monte Carlo (MCMC) sampling was performed with four chains, 5,000 adaptation iterations, and 100,000 total iterations, with thinning every 10 iterations. Inconsistency in the network was evaluated through node splitting analysis. Surface Under the Cumulative Ranking Curve (SUCRA) values were calculated to rank treatments by efficacy, with lower SUCRA values indicating less favorable outcomes.

**eTable 1. Quality Rating Scheme for the Included Studies**

|  | Quality Rating Scheme for Studies and Other Evidence |
| --- | --- |
| MSCRG (1996)^13,14^ | 1 |
| IFNβ-1b European Study Group (1998)^3,15^ | 1 |
| AFFIRM (2006)^16,17^ | 1 |
| REGARD (2008)^18^ | 1 |
| CAMMS223 (2008)^4^ | 1 |
| BEYOND (2009)^19^ | 1 |
| Montalban et al. (2009)^5^ | 1 |
| FREEDOMS I (2010)^20^ | 1 |
| TRANSFORMS (2010)^21^ | 1 |
| TEMSO (2011)^1,2^ | 1 |
| CONFIRM (2012)^22,23^ | 1 |
| CARE-MS I (2012)^24^ | 1 |
| CARE-MS II (2012)^25^ | 1 |
| GALA (2013)^26^ | 1 |
| DEFINE (2014)^27,28^ | 1 |
| ADVANCE (2014)^29^ | 1 |
| FREEDOMS II (2014)^30^ | 1 |
| INFORMS (2016)^31^ | 1 |
| OPERA I (2017)^32^ | 1 |
| OPERA II (2017)^32^ | 1 |
| ORATORIO (2017)^6^ | 1 |
| CLARITY (2018)^33,34^ | 1 |
| EXPAND (2018)^35^ | 1 |
| ASCEND (2018)^36^ | 1 |
| SUNBEAM (2019)^10^ | 1 |
| RADIANCE (2019)^9^ | 1 |
| ASCLEPIOS I (2020)^37^ | 1 |
| ASCLEPIOS II (2020)^37^ | 1 |
| ASSESS (2020)^7^ | 1 |
| OPTIMUM (2021)^38^ | 1 |
| FUMAPMS (2021)^8^ | 1 |
| ULTIMATE I (2022)^11^ | 1 |
| ULTIMATE II (2022)^11^ | 1 |

*A quality score of 1 reflects a properly powered and conducted randomized clinical trial.*

**eTable 2. Sensitivity Analyses on the Association Between Treatment Effects on Brain Volume Loss and Disability Progression**

|  | Atrophy | | Lesions | | Model R^2^ |
| --- | --- | --- | --- | --- | --- |
|  | Beta | p-value | Beta | p-value |  |
| Excluding MSCRG (1996)^13,14^ | 0.388 | **0.0101** | 0.626 | **0.0002** | 0.560 |
| Excluding IFNβ-1b European Study Group (1998)^3,15^ | 0.422 | **0.0047** | 0.620 | **0.0001** | 0.560 |
| Excluding AFFIRM (2006)^16,17^ | 0.413 | **0.0084** | 0.629 | **0.0002** | 0.530 |
| Excluding REGARD (2008)^18^ | 0.413 | **0.0098** | 0.608 | **0.0004** | 0.508 |
| Excluding CAMMS223 (2008)^4^ | 0.422 | **0.0047** | 0.620 | **0.0001** | 0.560 |
| Excluding BEYOND (2009)^19^ | 0.434 | **0.0069** | 0.616 | **0.0003** | 0.513 |
| Excluding Montalban et al. (2009)^5^ | 0.422 | **0.0047** | 0.620 | **0.0001** | 0.560 |
| Excluding FREEDOMS I (2010)^20^ | 0.406 | **0.0083** | 0.628 | **0.0002** | 0.546 |
| Excluding TRANSFORMS (2010)^21^ | 0.405 | **0.0070** | 0.628 | **0.0001** | 0.569 |
| Excluding TEMSO (2011)^1,2^ | 0.417 | **0.0063** | 0.622 | **0.0002** | 0.558 |
| Excluding CONFIRM (2012)^22,23^ | 0.463 | **0.0051** | 0.598 | **0.0006** | 0.539 |
| Excluding CARE-MS I (2012)^24^ | 0.390 | **0.0090** | 0.634 | **0.0001** | 0.572 |
| Excluding CARE-MS II (2012)^25^ | 0.408 | **0.0057** | 0.647 | **<0.0001** | 0.587 |
| Excluding GALA (2013)^26^ | 0.412 | **0.0092** | 0.612 | **0.0003** | 0.518 |
| Excluding DEFINE (2014)^27,28^ | 0.427 | **0.0059** | 0.611 | **0.0002** | 0.545 |
| Excluding ADVANCE (2014)^29^ | 0.479 | **0.0010** | 0.632 | **<0.0001** | 0.626 |
| Excluding FREEDOMS II (2014)^30^ | 0.422 | **0.0057** | 0.621 | **0.0002** | 0.558 |
| Excluding INFORMS (2016)^31^ | 0.412 | **0.0065** | 0.626 | **0.0001** | 0.564 |
| Excluding OPERA I (2017)^32^ | 0.422 | **0.0057** | 0.620 | **0.0002** | 0.558 |
| Excluding OPERA II (2017)^32^ | 0.428 | **0.0055** | 0.609 | **0.0002** | 0.552 |
| Excluding ORATORIO (2017)^6^ | 0.422 | **0.0047** | 0.620 | **0.0001** | 0.560 |
| Excluding CLARITY (2018)^33,34^ | 0.422 | **0.0002** | 0.620 | **0.0057** | 0.560 |
| Excluding EXPAND (2018)^35^ | 0.454 | **0.0027** | 0.569 | **0.0003** | 0.585 |
| Excluding ASCEND (2018)^36^ | 0.411 | **0.0049** | 0.652 | **<0.0001** | 0.601 |
| Excluding SUNBEAM (2019)^10^ | 0.422 | **0.0047** | 0.620 | **0.0001** | 0.560 |
|  |  |  |  |  |  |
| Excluding RADIANCE (2019)^9^ | 0.508 | **0.0005** | 0.605 | **<0.0001** | 0.632 |
| Excluding ASCLEPIOS I (2020)^37^ | 0.428 | **0.0058** | 0.607 | **0.0003** | 0.545 |
| Excluding ASCLEPIOS II (2020)^37^ | 0.425 | **0.0047** | 0.632 | **0.0001** | 0.575 |
| Excluding ASSESS (2020)^7^ | 0.422 | **0.0047** | 0.620 | **0.0001** | 0.560 |
| Excluding OPTIMUM (2021)^38^ | 0.424 | **0.0055** | 0.612 | **0.0002** | 0.561 |
| Excluding FUMAPMS (2021)^8^ | 0.422 | **0.0047** | 0.620 | **0.0001** | 0.560 |
| Excluding ULTIMATE I (2022)^11^ | 0.354 | **0.0171** | 0.622 | **0.0002** | 0.571 |
| Excluding ULTIMATE II (2022)^11^ | 0.422 | **0.0047** | 0.620 | **0.0001** | 0.560 |
| Unweighted regression | 0.518 | **0.0013** | 0.587 | **0.0004** | 0.528 |
| Accounting for atrophy software* | 0.454 | **0.0015** | 0.504 | **0.0011** | 0.640 |
| Excluding PMS | 0.413 | **0.0065** | 0.607 | **0.0002** | 0.619 |
| Accounting for age, sex, EDSS | 0.367 | **0.0399** | 0.663 | **0.0006** | 0.570 |
| Considering BVL from the longest observation period | 0.393 | **0.0094** | 0.595 | **0.0003** | 0.536 |

*Analysis conducted incorporating software (as a binary variable: SIENA vs others) as an additional covariate in the weighted linear regression model. ** Analysis conducted incorporating age, sex, and EDSS in the treatment arm as additional covariates in the weighted linear regression model.
Abbreviations: BVL, brain volume loss; EDSS, Expanded Disability Status Scale, PMS, progressive multiple sclerosis

**eTable 3. Sensitivity Analyses on the Association Between Treatment Effects on Brain Volume Loss and Disability – Univariate Associations**

|  | Beta | p-value | R^2^ |
| --- | --- | --- | --- |
| Entire cohort (n=31) | 0.466 | 0.0083 | 0.217 |
| a) Follow-up ≥24 months (n=24) | 0.491 | 0.0148 | 0.207 |
| b) In RMS (n=25) | 0.545 | 0.0049 | 0.266 |
| c) In RRMS (n=21) | 0.680 | 0.0007 | 0.435 |
| d) Using SIENA or BPF (n=24) | 0.675 | 0.0003 | 0.431 |
| e) Only with rebaseline | 0.738 | 0.0040 | 0.503 |
| a) + b) + d) (n=15) | 0.733 | 0.0019 | 0.501 |
| a) + c) + d) (n=14) | 0.852 | 0.0001 | 0.703 |
| a) + b) + e) (n=10) | 0.758 | 0.0111 | 0.521 |
| a) + b) + d) + e) (n=9) | 0.650 | 0.0579 | 0.341 |
| a) + c) + d) + e) (n=9) | 0.650 | 0.0579 | 0.341 |

Associations were explored using univariate linear regression. n reflect the number of included studies.
*Abbreviations: β, standardized regression coefficient; BPF, brain parenchymal fraction; RMS, relapsing multiple sclerosis; RRMS, relapsing-remitting multiple sclerosis; SIENA, Structural Image Evaluation, using Normalisation, of Atrophy.*

**eTable 4. Meta-regression: Association between treatment effect on BVL and treatment effect on disability accumulation (with 3-month confirmation), accounting for treatment effect on MRI lesions – Weighted regression model using treatment estimates derived from original RCTs**

|  | Estimate | SE | p-value |
| --- | --- | --- | --- |
| Intercept | 0.0771 | 0.0564 | 0.1810 |
| Treatment effect on MRI lesion accumulation | 0.2281 | 0.0394 | <0.0001 |
| Treatment effect on BVL | 0.3255 | 0.0979 | 0.0022 |
| R^2^=0.667 | | | |

Abbreviations: BVL, brain volume loss; MRI, magnetic resonance imaging; SE, standard error.

**eTable 5. Meta-regression: Association between treatment effect on BVL and treatment effect on disability accumulation (with 3-month confirmation), accounting for treatment effect on MRI lesions – Weighted regression model using treatment estimates from the NMAs**

|  | Estimate | SE | p-value |
| --- | --- | --- | --- |
| Intercept | 0.0499 | 0.0772 | 0.5227 |
| Treatment effect on MRI lesion accumulation | 0.1884 | 0.0464 | 0.0003 |
| Treatment effect on BVL | 0.4414 | 0.1658 | 0.0121 |
| R^2^=0.387 | | | |

Abbreviations: BVL, brain volume loss; MRI, magnetic resonance imaging; SE, standard error.

**eTable 6. Characteristics of RCTs reporting BVL Estimates**

| **Trial** | **Experimental arm** | **Control**  **arm** | **Longest BVL observation period** | **Longest BVL observation period with rebaseline** | **BVL Approach** |
| --- | --- | --- | --- | --- | --- |
| MSCRG (1996) | Interferon β-1a | Placebo | 0-24 months | 12-24 months | BPF |
| IFNβ-1b European Study Group (1998) | Interferon β-1b | Placebo | 0-36 months | / | Not specified |
| AFFIRM (2006) | Natalizumab | Placebo | 0-24 months | 12-24 months | BPF |
| REGARD (2008) | Interferon β-1a | Glatiramer acetate | 0-96 weeks | / | SIENA |
| CAMMS223 (2008) | Alemtuzumab | Interferon β-1a | 0-36 months | 12-36 months | Not specified |
| BEYOND (2009) | Interferon β-1b | Glatiramer acetate | 0-up to 42 months | / | SIENA |
| Montalban et al. (2009) | Interferon β-1b | Placebo | 0-24 months | 12-24 months | BPF |
| FREEDOMS I (2010) | Fingolimod | Placebo | 0-24 months | 12-24 months | SIENA |
| TRANSFORMS (2010) | Fingolimod | Interferon β-1a | 0-12 months | / | SIENA |
| TEMSO (2011) | Teriflunomide | Placebo | 0-24 months | / | SIENA |
| CONFIRM (2012) | Dimethyl fumarate  Glatiramer acetate | Placebo | 0-24 months | 12-24 months | SIENA |
| CARE-MS I (2012) | Alemtuzumab | Interferon β-1a | 0-24 months | / | BPF |
| CARE-MS II (2012) | Alemtuzumab | Interferon β-1a | 0-24 months | / | BPF |
| GALA (2013) | Glatiramer acetate | Placebo | 0-12 months | / | SIENA |
| DEFINE (2014) | Dimethyl fumarate | Placebo | 0-96 weeks | 24-96 weeks | SIENA |
| ADVANCE (2014) | Peginterferon β-1a | Placebo | 0-12 months | / | Not specified |
| FREEDOMS II (2014) | Fingolimod | Placebo | 0-24 months | 12-24 months | SIENA |
| INFORMS (2016) | Fingolimod | Placebo | 0-36 months | / | SIENA |
| OPERA I (2017) | Ocrelizumab | Interferon β-1a | 24-96 months | 24-96 months | SIENA |
| OPERA II (2017) | Ocrelizumab | Interferon β-1a | 24-96 months | 24-96 months | SIENA |
| ORATORIO (2017) | Ocrelizumab | Placebo | 24-120 weeks | 24-120 weeks | SIENA |
| CLARITY (2018) | Cladribine | Placebo | 6-24 months | 6-24 months | SIENA |
| EXPAND (2018) | Siponimod | Placebo | 0-24 months | 12-24 months | Not specified |
| ASCEND (2018) | Natalizumab | Placebo | 24-96 weeks | 24-96 weeks | SIENA |
| SUNBEAM (2019) | Ozanimod | Interferon β-1a | 0-12 months | / | SIENA |
| RADIANCE (2019) | Ozanimod | Interferon β-1a | 0-24 months | / | SIENA |
| ASCLEPIOS I (2020) | Ofatumumab | Teriflunomide | 0-30 months | / | Not specified |
| ASCLEPIOS II (2020) | Ofatumumab | Teriflunomide | 0-30 months | / | Not specified |
| ASSESS (2020) | Fingolimod | Glatiramer acetate | 0-12 months | / | Not specified |
| OPTIMUM (2021) | Ponesimod | Teriflunomide | 0-108 weeks | / | SIENA |
| FUMAPMS (2021) | Dimethyl fumarate | Placebo | 0-48 weeks | / | Not specified |
| ULTIMATE I (2022) | Ublituximab | Teriflunomide | 0-96 weeks | / | Not specified |
| ULTIMATE II (2022) | Ublituximab | Teriflunomide | 0-96 weeks | / | Not specified |

*Abbreviations: BPF, brain parenchymal fraction.*

***eTable 7. Causal mediation analysis – Mediation of the relationship between treatment effect on BVL and treatment effect on disability accumulation by treatment effect on MRI lesion accumulation***

| *Term* | *Estimate* | *95% CI* | *p-value* |
| --- | --- | --- | --- |
| *ACME* | *-0.002* | *[-0.23; 0.32]* | *0.996* |
| *ADE* | *0.418* | *[0.16; 0.65]* | *0.008* |
| *Total Effect* | *0.416* | *[0.09; 0.82]* | *0.014* |
| *Proportion Mediated* | *-0.005* | *[-1.60; 0.54]* | *0.990* |

*Abbreviations: ACME, Average Causal Mediation Effect; ADE, Average Direct Effect; CI, confidence interval.*

**eFigure 1. Comparison of Direct and Indirect Estimates in Network Meta-Analysis for Brain Volume Loss**


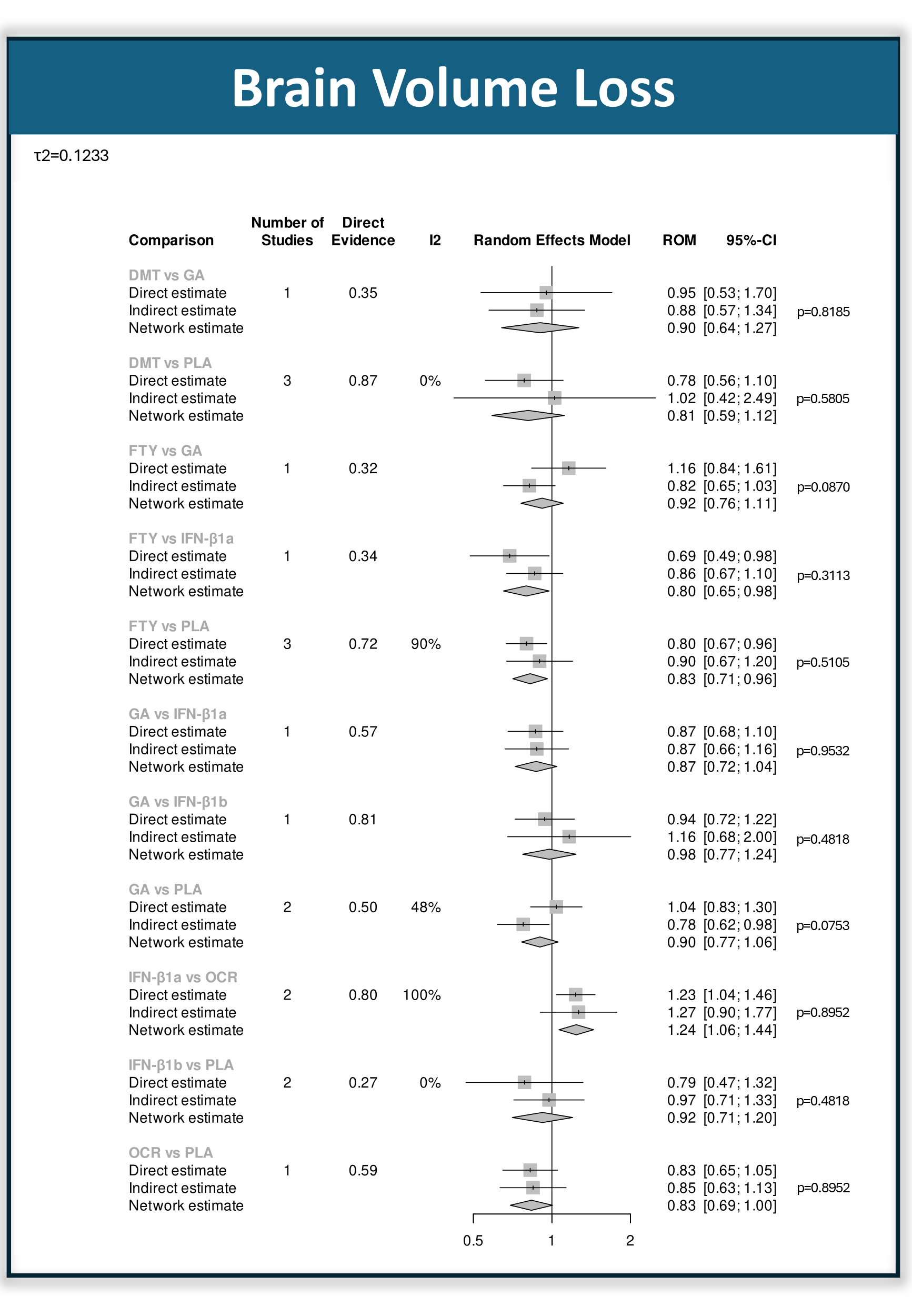


**eFigure 2. Comparison of Direct and Indirect Estimates in Network Meta-Analysis for MRI Lesion Activity**


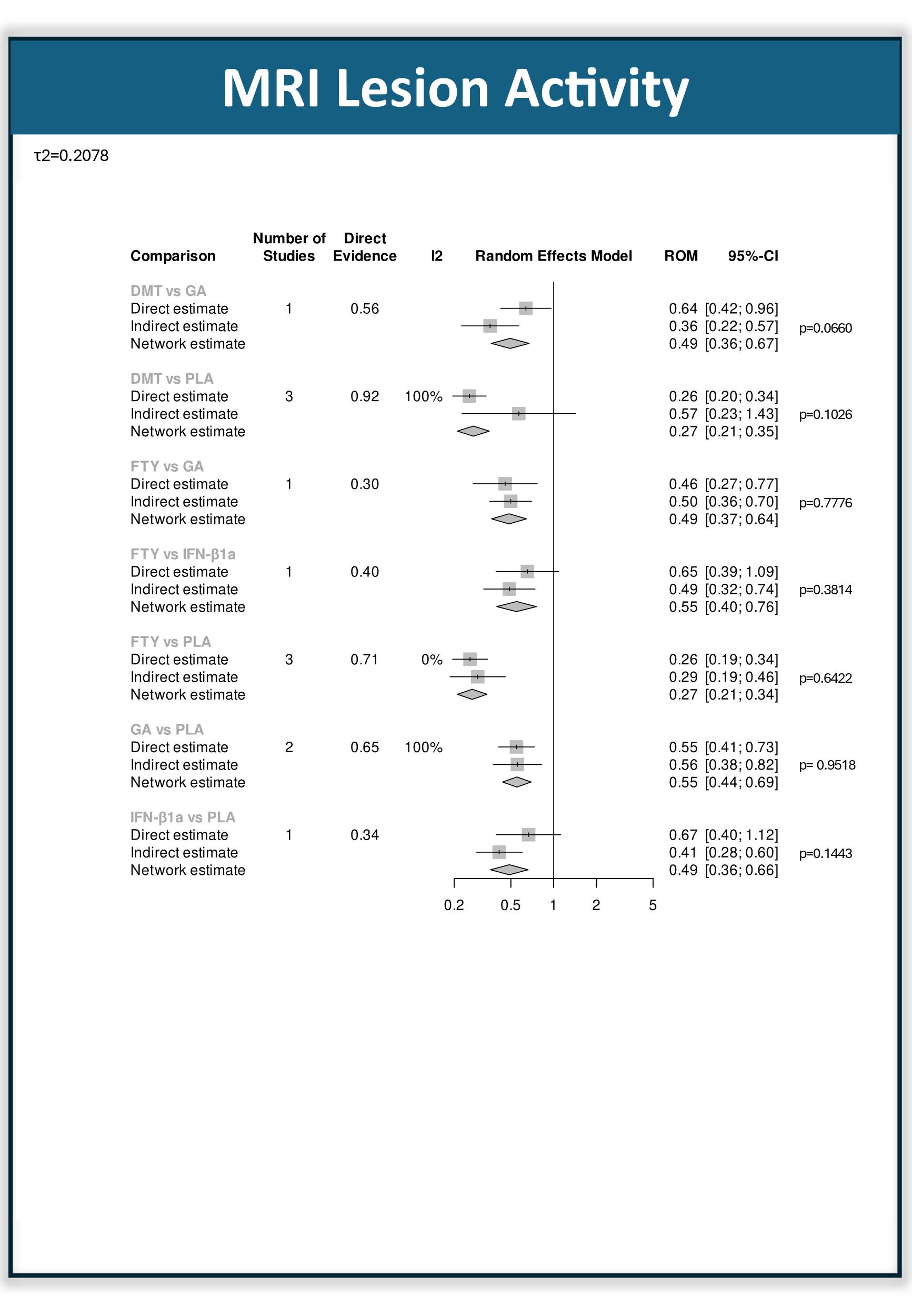


**eFigure 3. Comparison of Direct and Indirect Estimates in Network Meta-Analysis for Disability Progression**


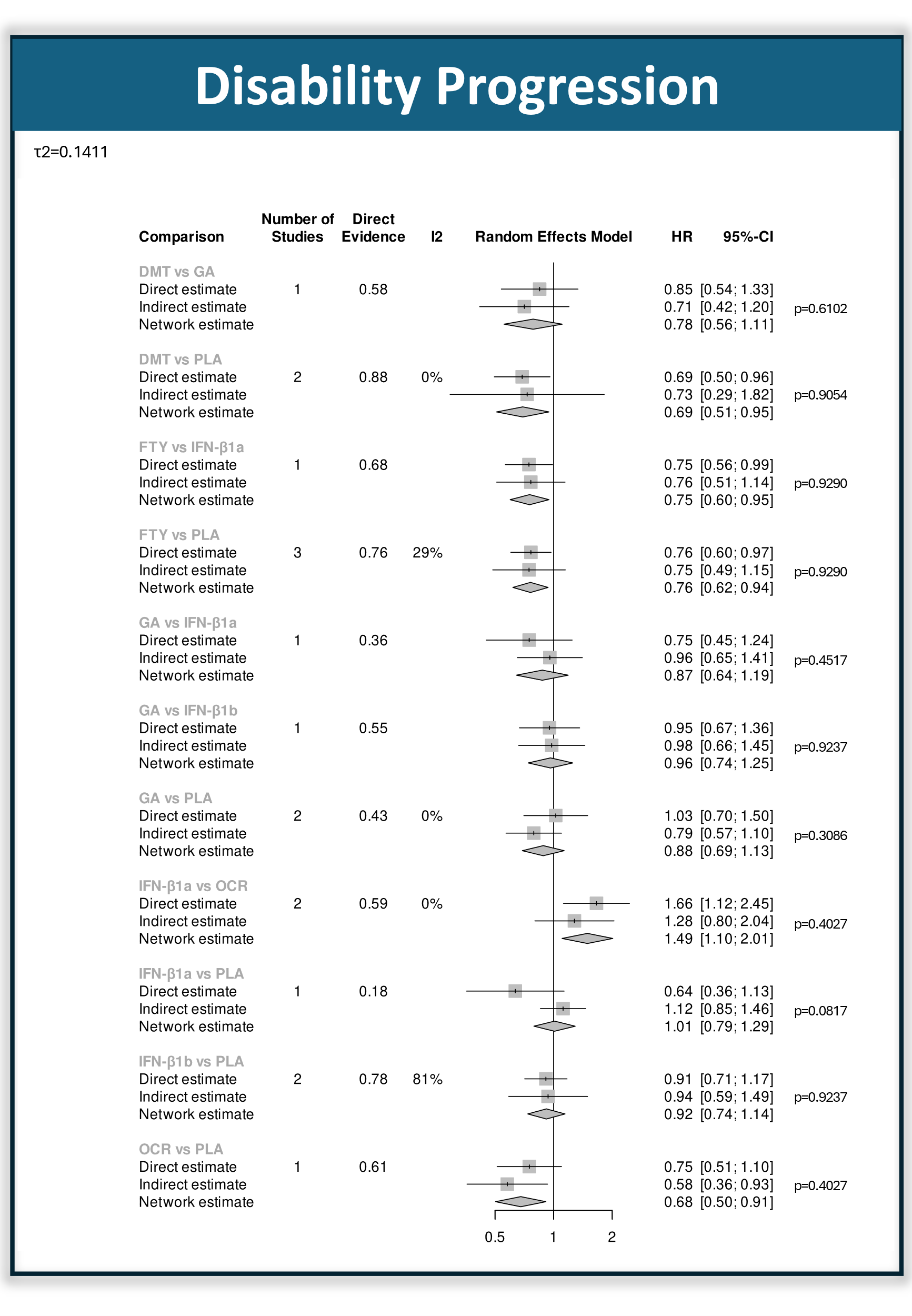


**eFigure 4. Funnel Plot – Brain Volume Loss**


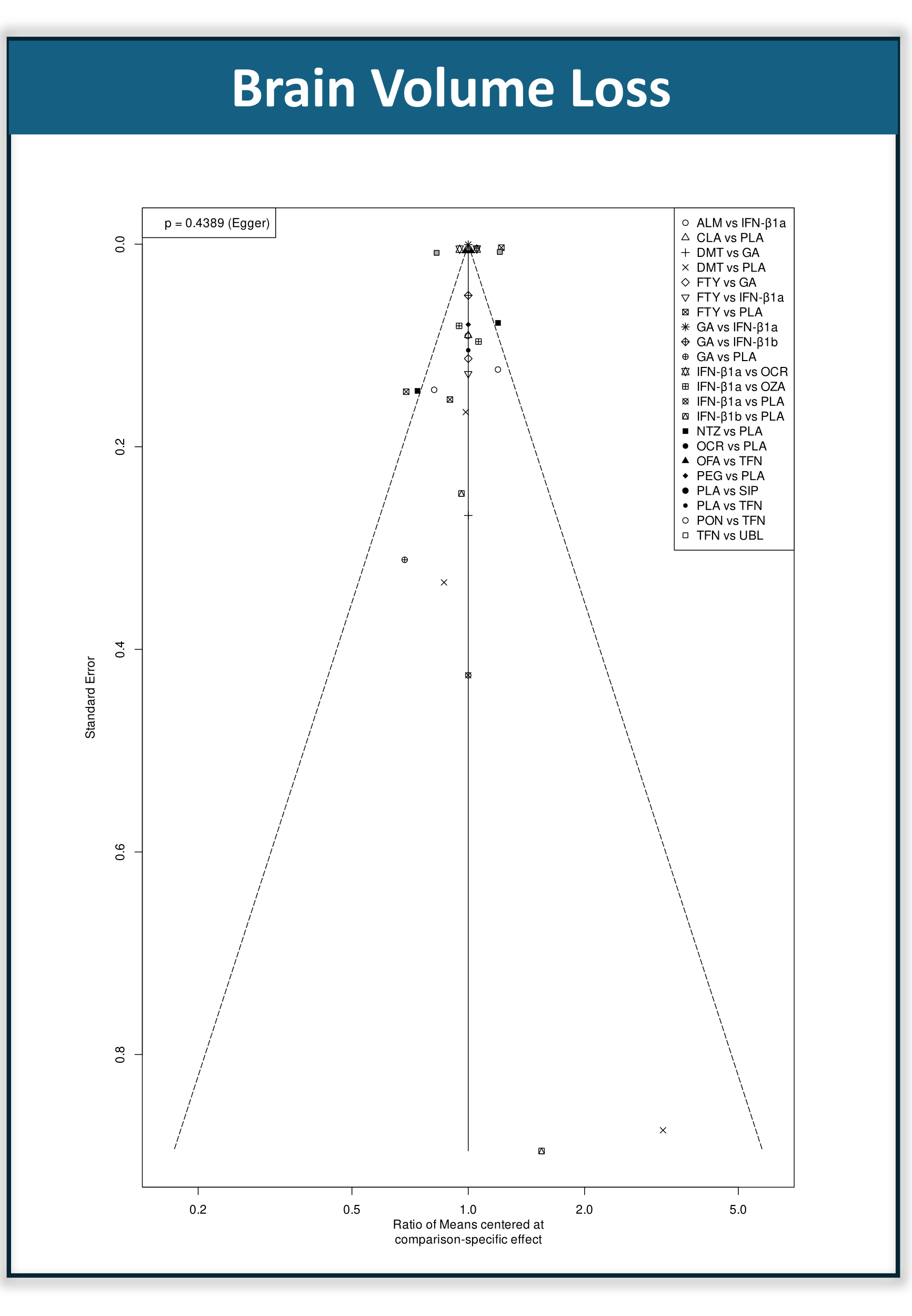


**eFigure 5. Funnel Plot – MRI Lesion Activity**


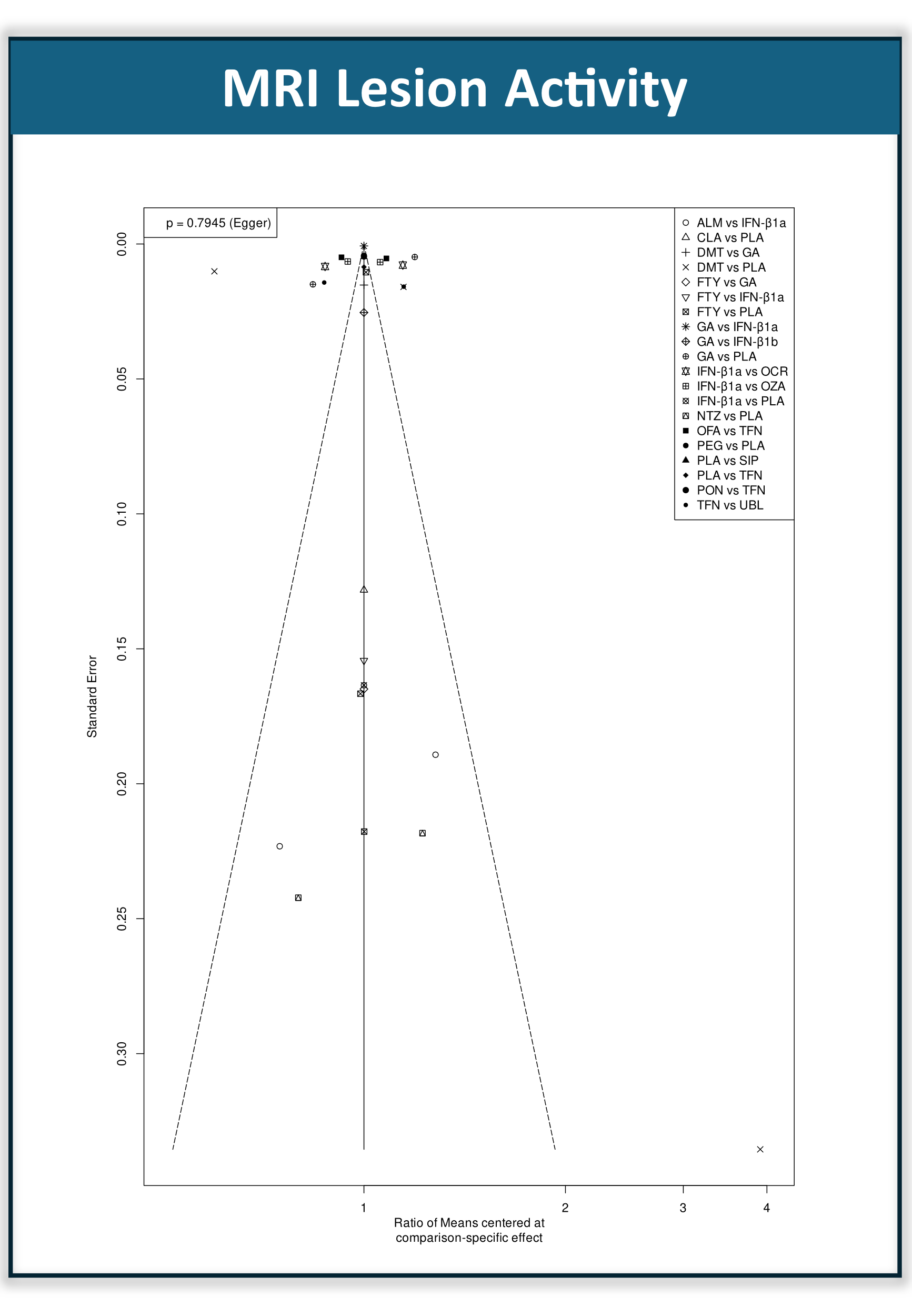


**eFigure 6. Funnel Plot – Disability Progression**


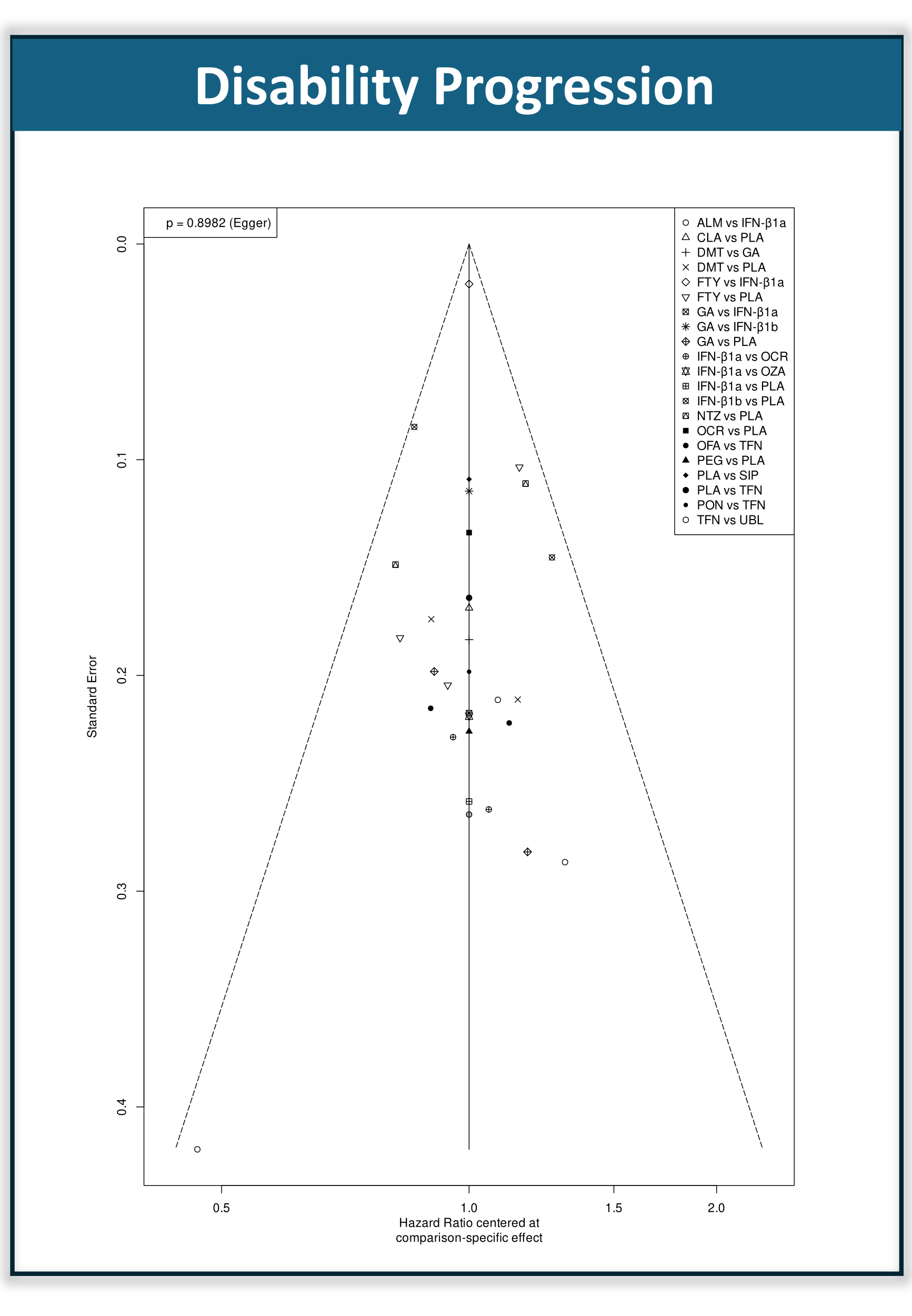


**eFigure 7. SUCRA Rankings from Bayesian Network Meta-Analysis of Treatment Effect on Brain Volume Loss**
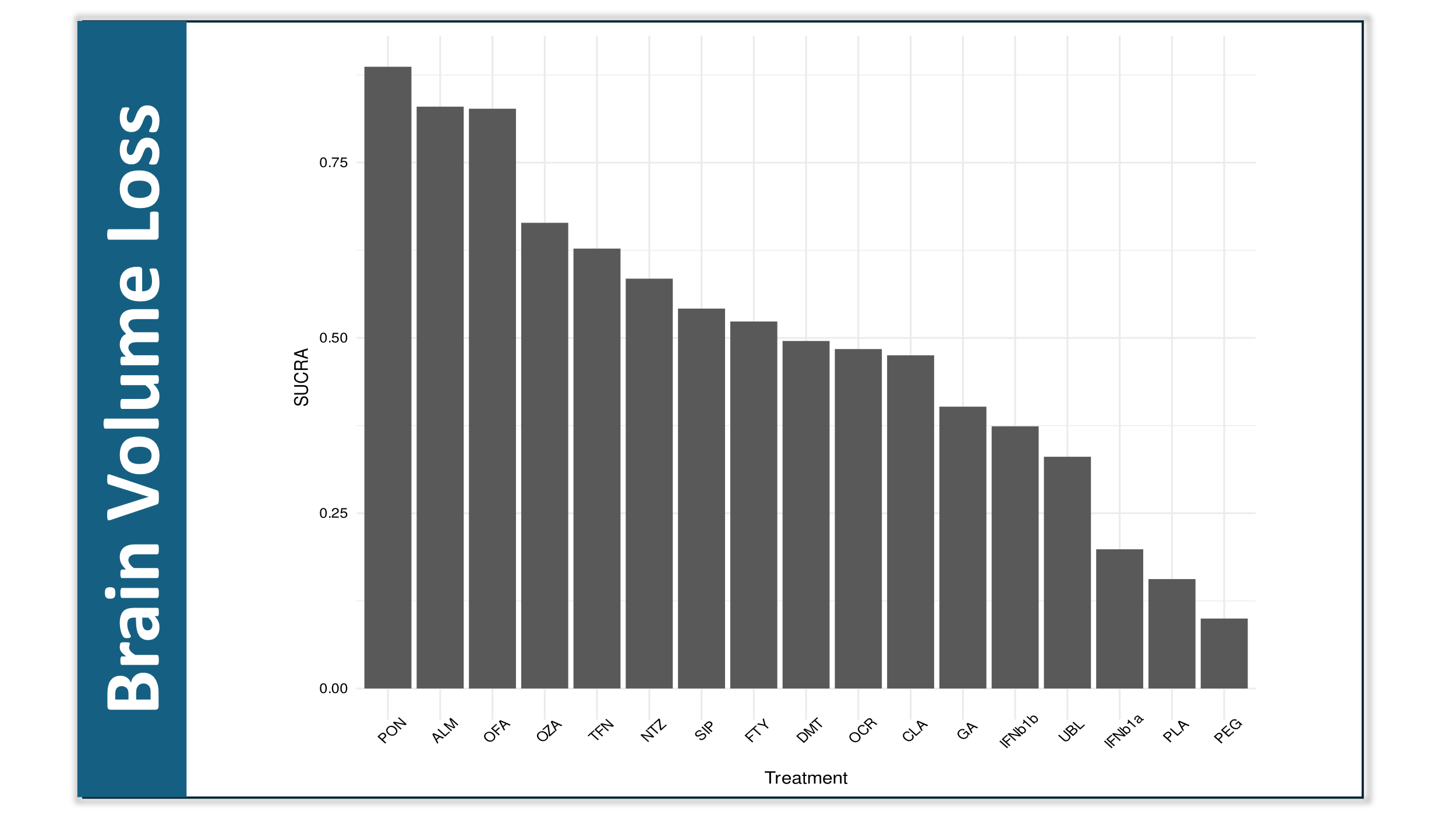


Abbreviations: ALM, alemtuzumab; CLA, cladribine; DMT, dimethyl fumarate; FTY, fingolimod; GA, glatiramer acetate; IFNb1a, interferon beta-1a; IFNb1b, interferon beta-1b; NTZ, natalizumab; OCR, ocrelizumab; OFA, ofatumumab; OZA, ozanimod; PEG, peginterferon beta-1a; PLA, placebo; PON, ponesimod; SIP, Siponimod; SUCRA, surface under the cumulative ranking curve; TFN, teriflunomide; UBL, ublituximab.

**eFigure 8. Sensitivity Analysis of Network Meta-Analysis Results: Treatment Effect on BVL Using the Longest Observation Period**


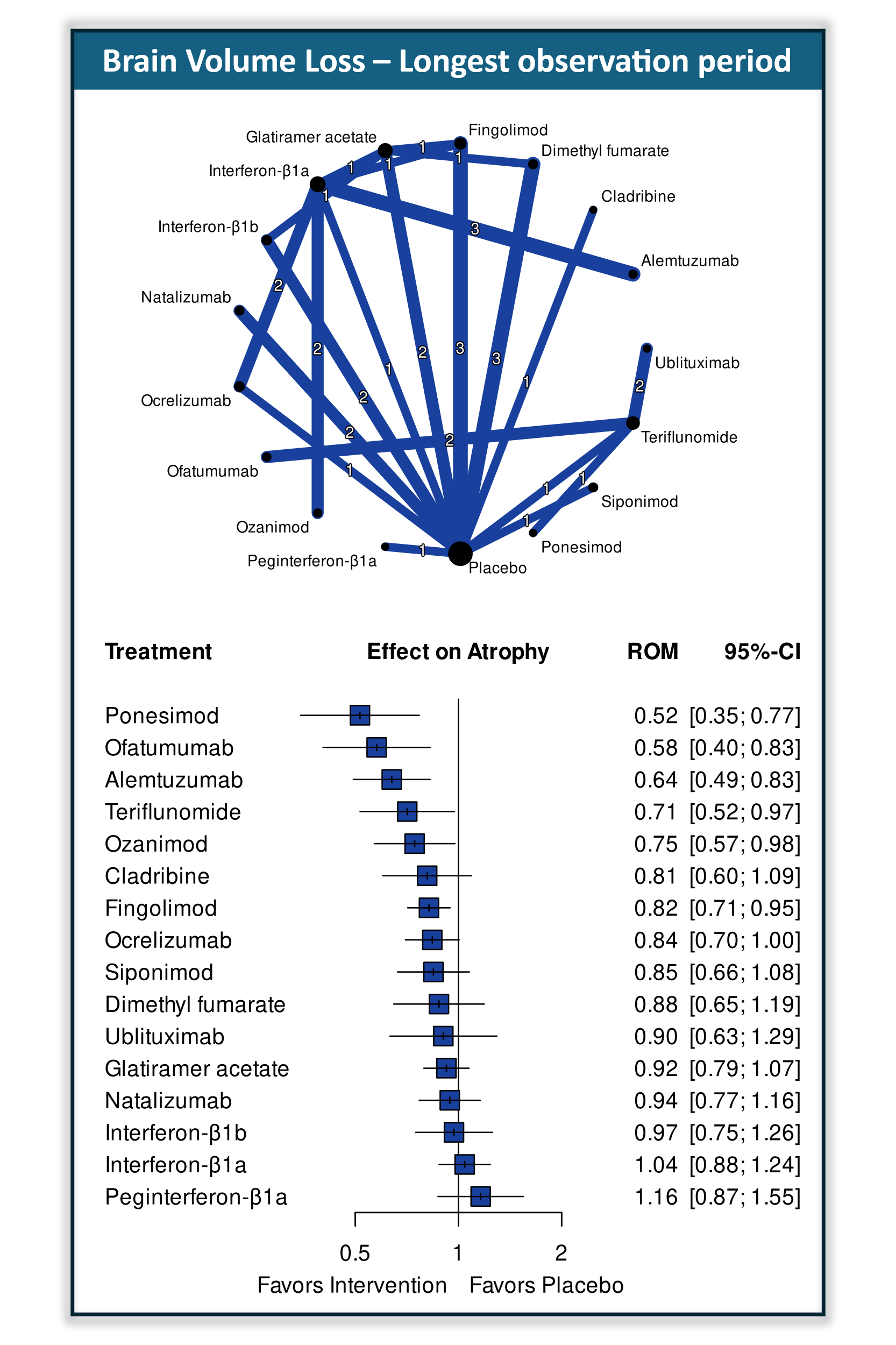


The figure includes two elements: 1) Top: A network diagram summarizing direct comparisons between disease-modifying treatments across randomized controlled trials. Each node represents a treatment, with node size proportional to the total number of participants receiving that treatment. Edges indicate direct comparisons, with thickness reflecting the number of contributing trials, and the exact number of trials labeled at the midpoint of each edge; 2) Bottom: Forest plots displaying treatment effects estimated from NMA models, compared to placebo.

**eFigure 9. Sensitivity Analysis of Network Meta-Analysis Results: Treatment Effect on BVL in RCTs conducted in RMS**


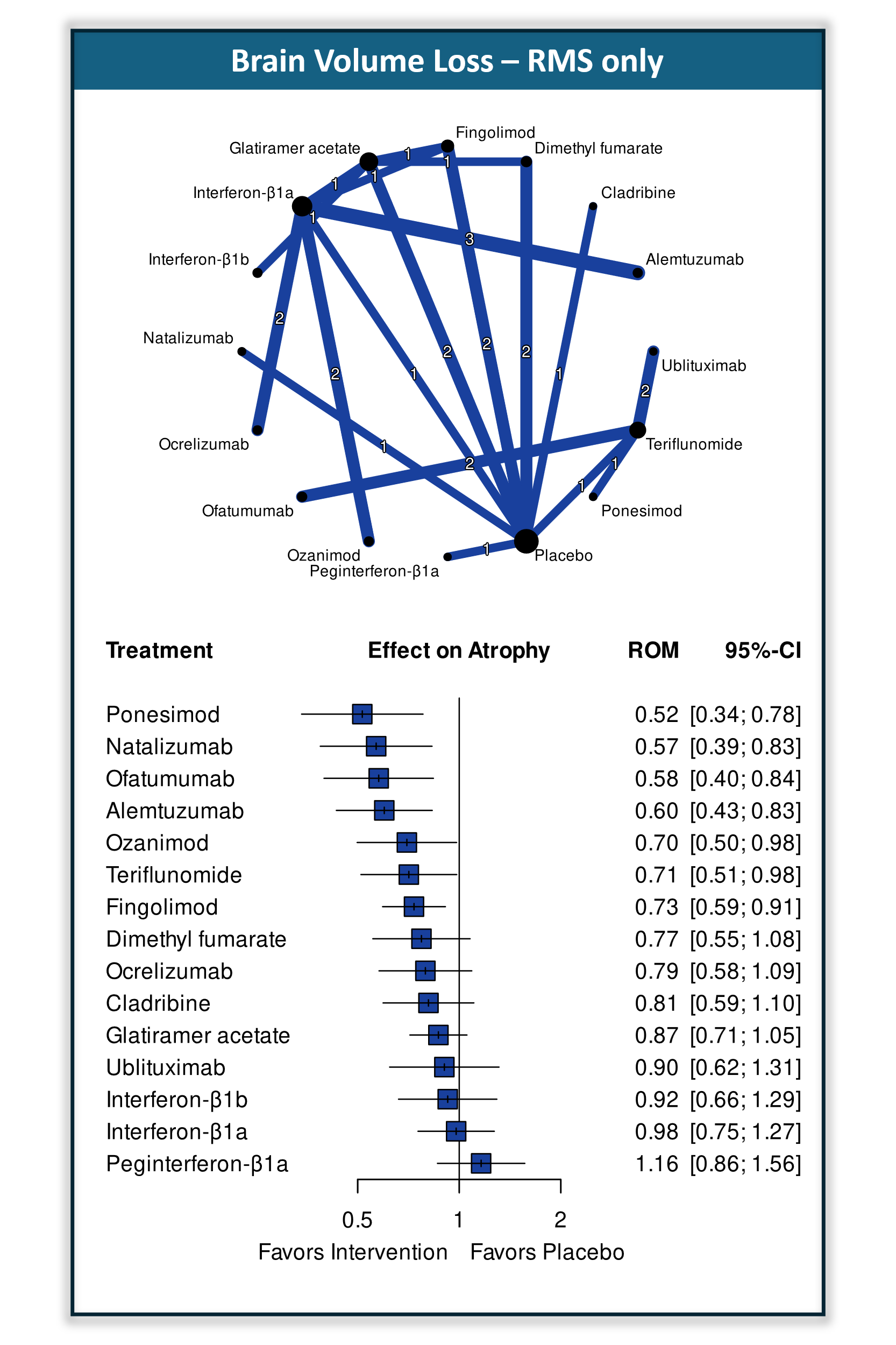


The figure includes two elements: 1) Top: A network diagram summarizing direct comparisons between disease-modifying treatments across randomized controlled trials. Each node represents a treatment, with node size proportional to the total number of participants receiving that treatment. Edges indicate direct comparisons, with thickness reflecting the number of contributing trials, and the exact number of trials labeled at the midpoint of each edge; 2) Bottom: Forest plots displaying treatment effects estimated from NMA models, compared to placebo.

**eFigure 10. Sensitivity Analysis of Network Meta-Analysis Results: Treatment Effect on BVL in RCTs using SIENA**


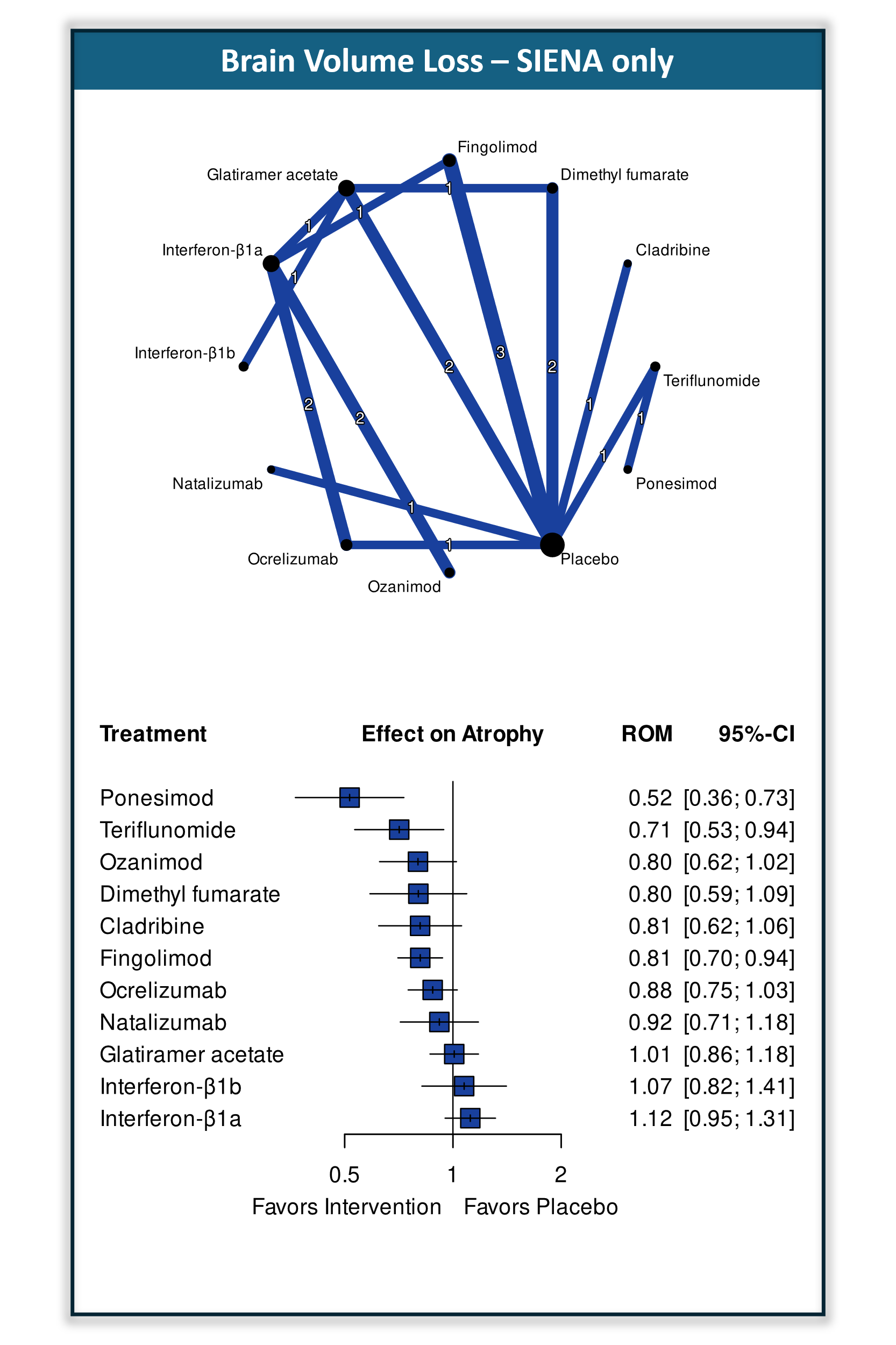


The figure includes two elements: 1) Top: A network diagram summarizing direct comparisons between disease-modifying treatments across randomized controlled trials. Each node represents a treatment, with node size proportional to the total number of participants receiving that treatment. Edges indicate direct comparisons, with thickness reflecting the number of contributing trials, and the exact number of trials labeled at the midpoint of each edge; 2) Bottom: Forest plots displaying treatment effects estimated from NMA models, compared to placebo.

**eFigure 11. Sensitivity Analysis of Network Meta-Analysis Results: Treatment Effect on BVL in RCTs With Rebaseline**


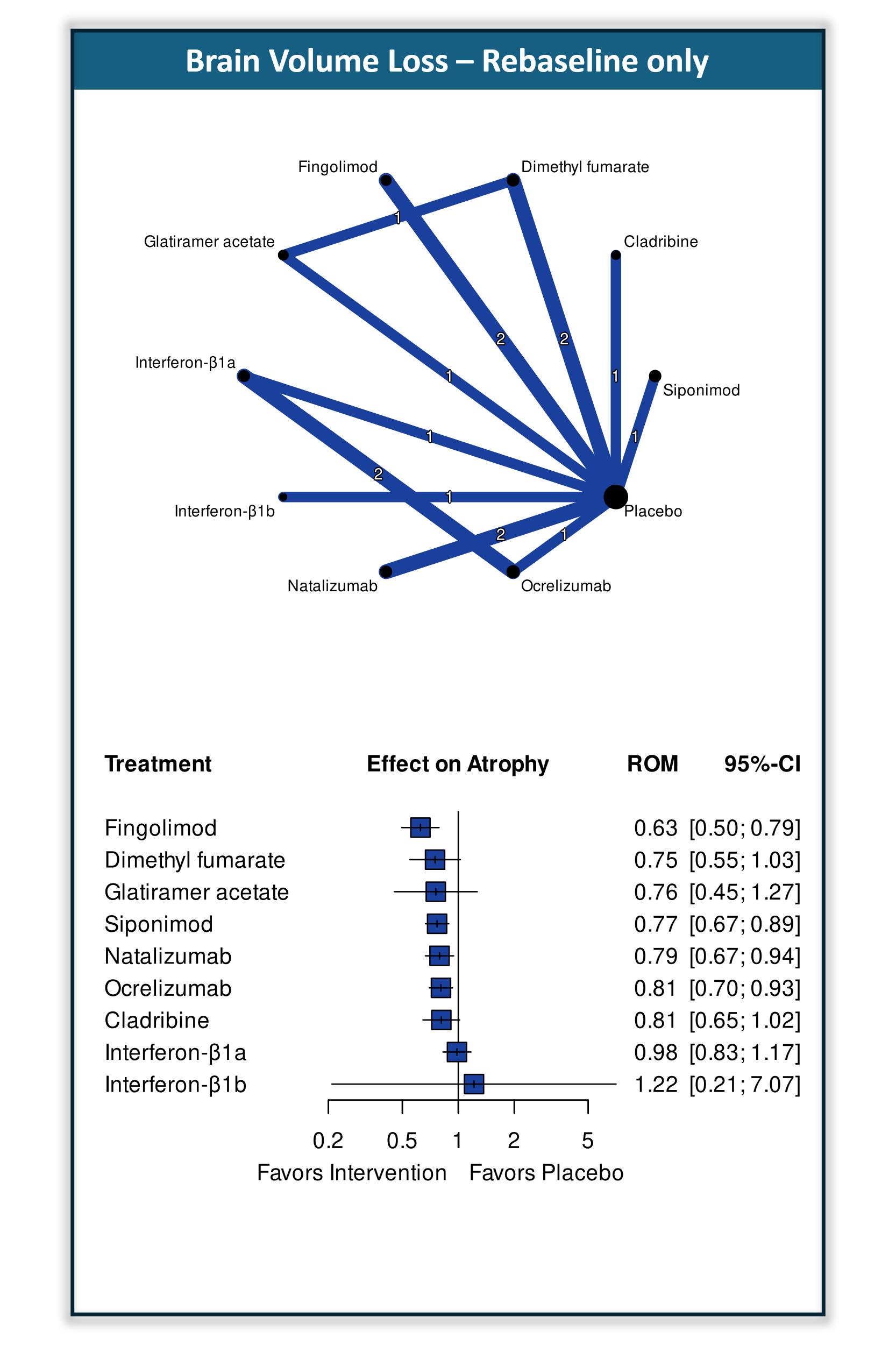


The figure includes two elements: 1) Top: A network diagram summarizing direct comparisons between disease-modifying treatments across randomized controlled trials. Each node represents a treatment, with node size proportional to the total number of participants receiving that treatment. Edges indicate direct comparisons, with thickness reflecting the number of contributing trials, and the exact number of trials labeled at the midpoint of each edge; 2) Bottom: Forest plots displaying treatment effects estimated from NMA models, compared to placebo.

**eFigure 12. Sensitivity Analysis of Network Meta-Analysis Results: Treatment Effect on BVL Excluding RCTs in Which Measures of Uncertainty Could Only Be Imputed**


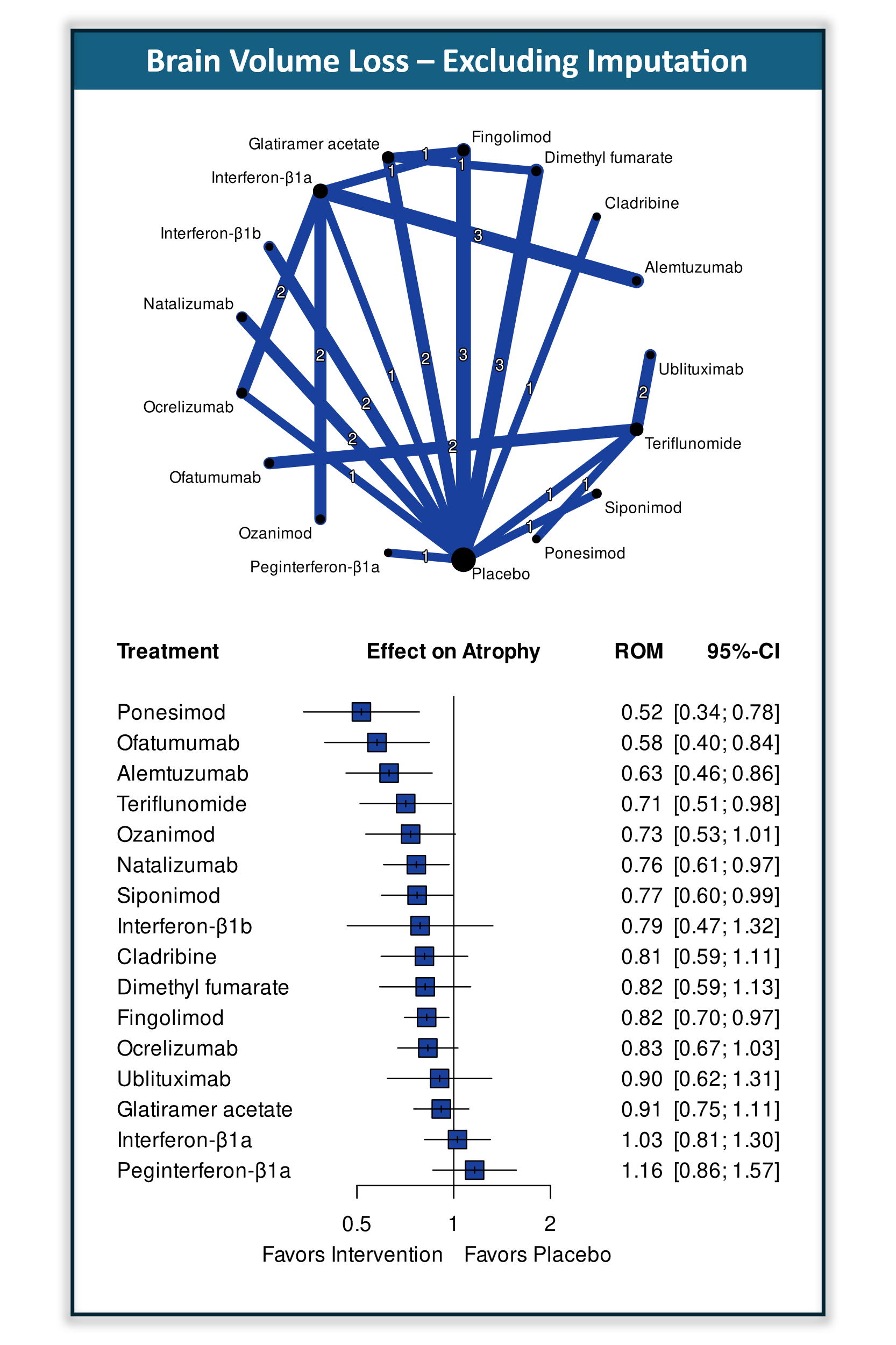


The figure includes two elements: 1) Top: A network diagram summarizing direct comparisons between disease-modifying treatments across randomized controlled trials. Each node represents a treatment, with node size proportional to the total number of participants receiving that treatment. Edges indicate direct comparisons, with thickness reflecting the number of contributing trials, and the exact number of trials labeled at the midpoint of each edge; 2) Bottom: Forest plots displaying treatment effects estimated from NMA models, compared to placebo.

**eFigure 13. Sensitivity Analysis of Network Meta-Analysis Results: Treatment Effect on MRI Lesion Activity Excluding RCTs in Which Measures of Uncertainty Could Only Be Imputed**


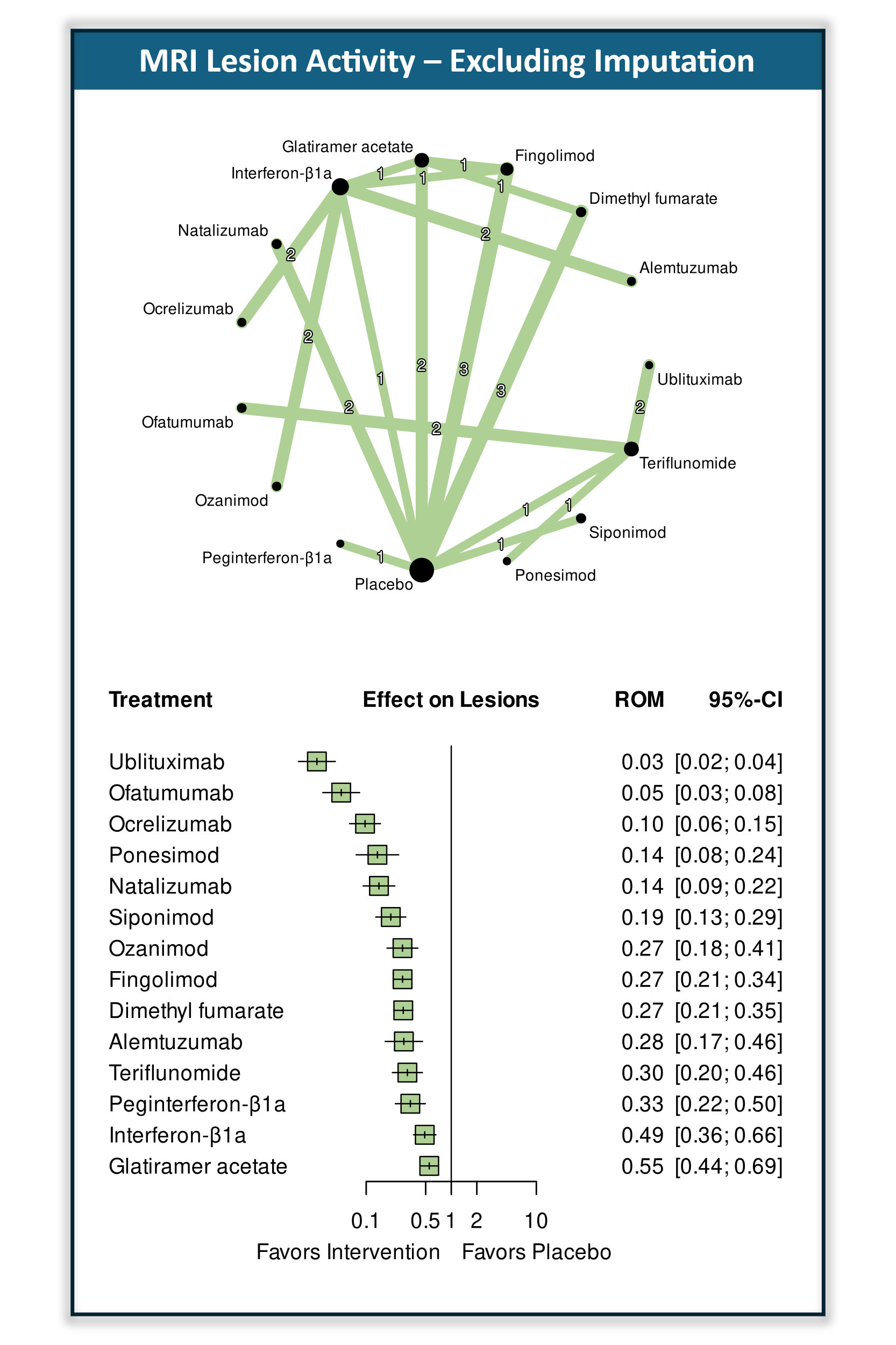


The figure includes two elements: 1) Top: A network diagram summarizing direct comparisons between disease-modifying treatments across randomized controlled trials. Each node represents a treatment, with node size proportional to the total number of participants receiving that treatment. Edges indicate direct comparisons, with thickness reflecting the number of contributing trials, and the exact number of trials labeled at the midpoint of each edge; 2) Bottom: Forest plots displaying treatment effects estimated from NMA models, compared to placebo.

**eFigure 14. Sensitivity Analysis of Network Meta-Analysis Results: Treatment Effect on Disability Progression Prioritizing Measures of Confirmed Disability Progression at Three Months**


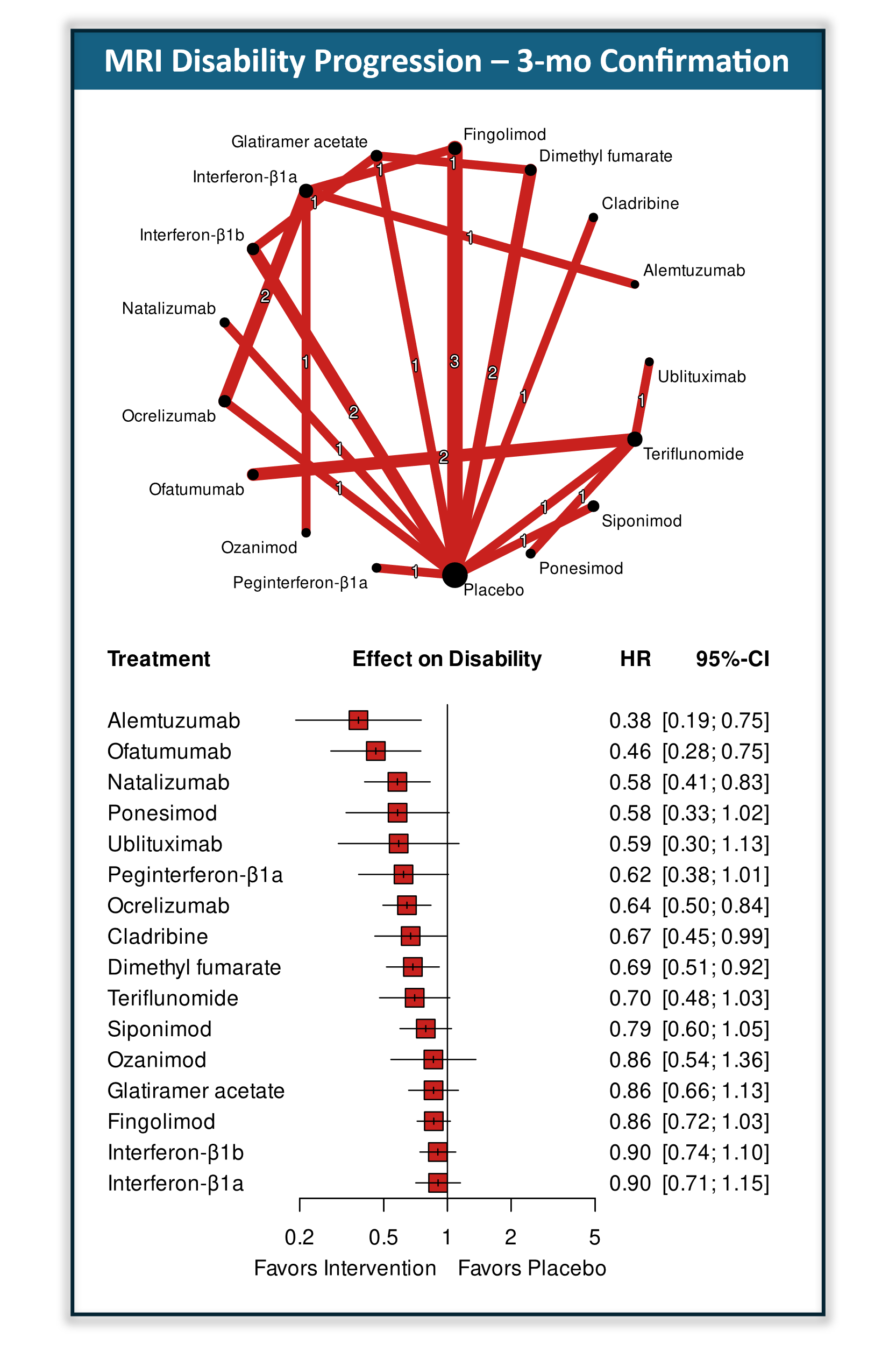


The figure includes two elements: 1) Top: A network diagram summarizing direct comparisons between disease-modifying treatments across randomized controlled trials. Each node represents a treatment, with node size proportional to the total number of participants receiving that treatment. Edges indicate direct comparisons, with thickness reflecting the number of contributing trials, and the exact number of trials labeled at the midpoint of each edge; 2) Bottom: Forest plots displaying treatment effects estimated from NMA models, compared to placebo.

**eFigure 15. Sensitivity Analysis of Network Meta-Analysis Results: Treatment Effect on BVL in RCTs conducted in RRMS**


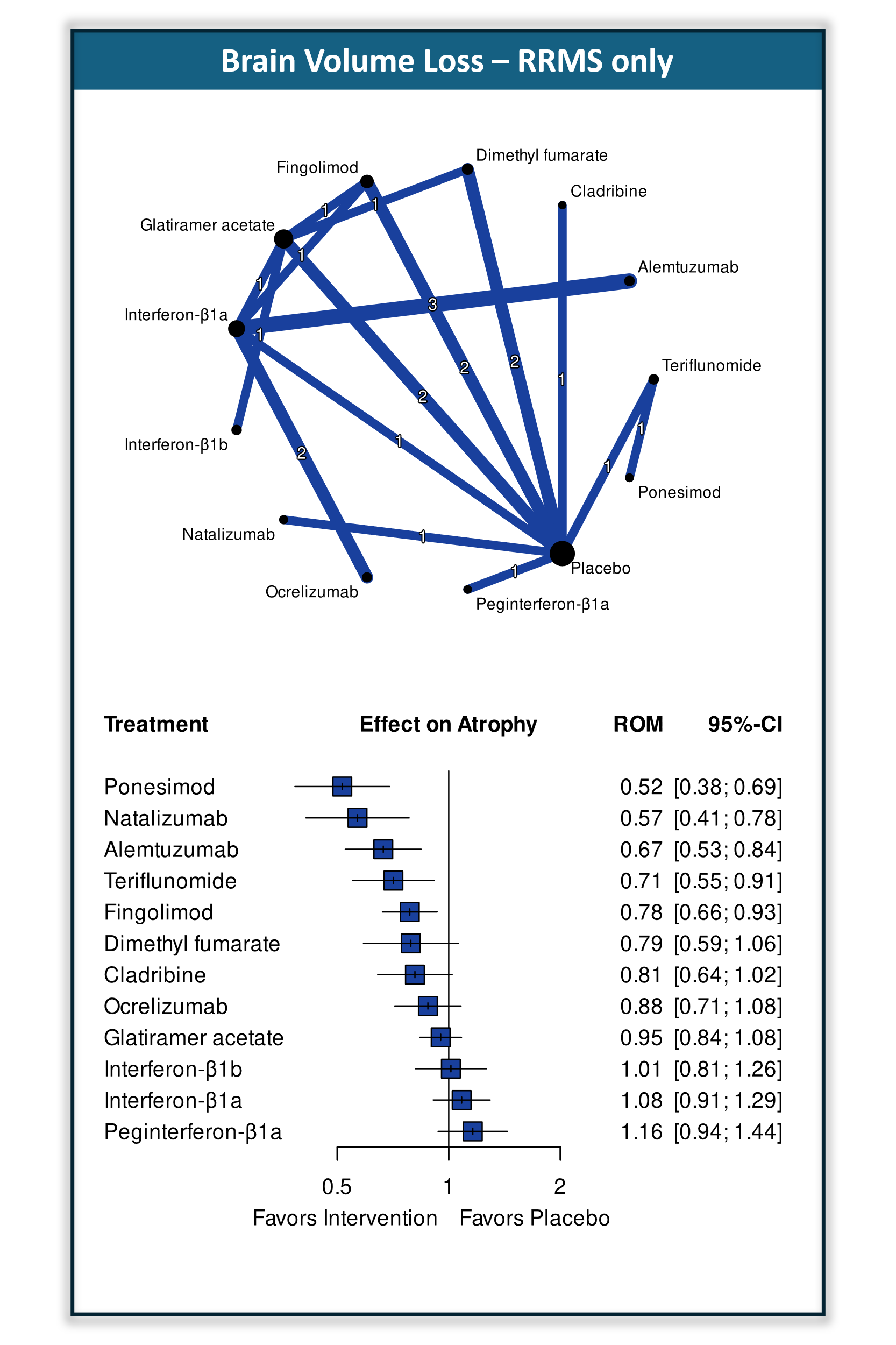


The figure includes two elements: 1) Top: A network diagram summarizing direct comparisons between disease-modifying treatments across randomized controlled trials. Each node represents a treatment, with node size proportional to the total number of participants receiving that treatment. Edges indicate direct comparisons, with thickness reflecting the number of contributing trials, and the exact number of trials labeled at the midpoint of each edge; 2) Bottom: Forest plots displaying treatment effects estimated from NMA models, compared to placebo.

**eFigure 16. SUCRA Rankings from Bayesian Network Meta-Analysis of Treatment Effect on MRI Lesion Activity**
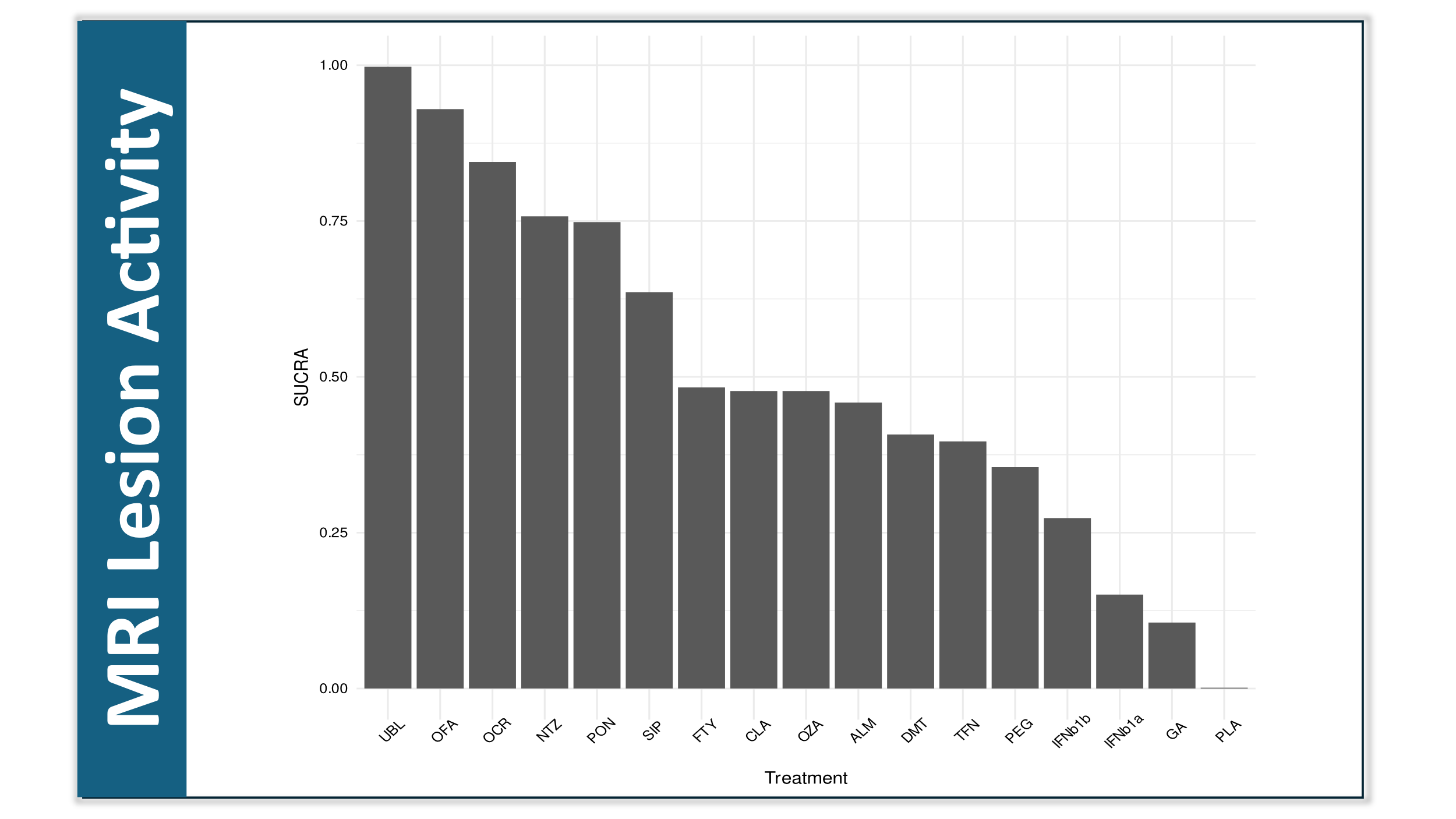


Abbreviations: ALM, alemtuzumab; CLA, cladribine; DMT, dimethyl fumarate; FTY, fingolimod; GA, glatiramer acetate; IFNb1a, interferon beta-1a; IFNb1b, interferon beta-1b; NTZ, natalizumab; OCR, ocrelizumab; OFA, ofatumumab; OZA, ozanimod; PEG, peginterferon beta-1a; PLA, placebo; PON, ponesimod; SIP, Siponimod; SUCRA, surface under the cumulative ranking curve; TFN, teriflunomide; UBL, ublituximab.

**eFigure 17. SUCRA Rankings from Bayesian Network Meta-Analysis of Treatment Effect on Disability Progression**


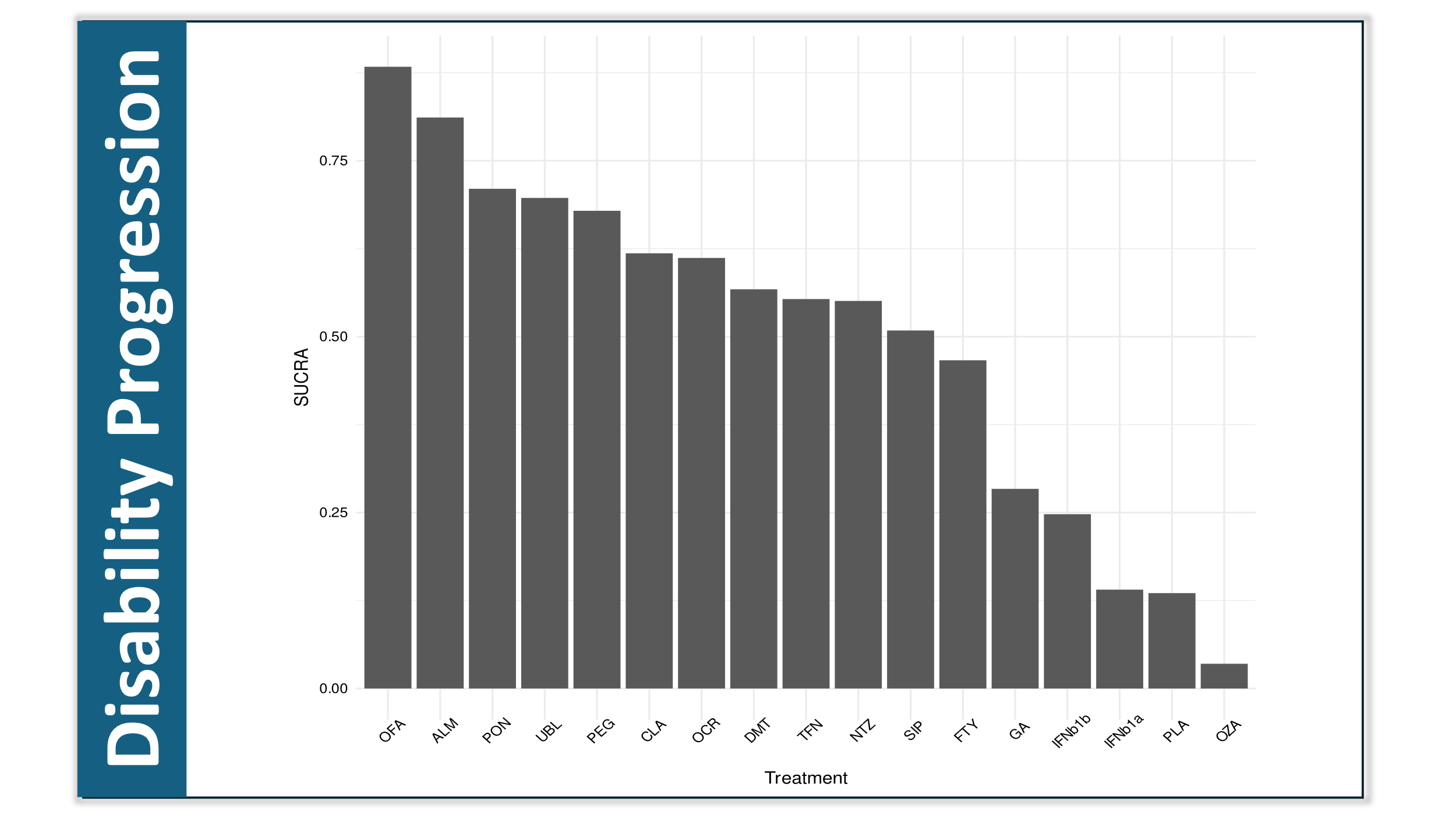


Abbreviations: ALM, alemtuzumab; CLA, cladribine; DMT, dimethyl fumarate; FTY, fingolimod; GA, glatiramer acetate; IFNb1a, interferon beta-1a; IFNb1b, interferon beta-1b; NTZ, natalizumab; OCR, ocrelizumab; OFA, ofatumumab; OZA, ozanimod; PEG, peginterferon beta-1a; PLA, placebo; PON, ponesimod; SIP, Siponimod; SUCRA, surface under the cumulative ranking curve; TFN, teriflunomide; UBL, ublituximab.
